# Author Correction: Systematic identification of post-transcriptional regulatory modules

**DOI:** 10.1038/s41467-024-52903-4

**Published:** 2024-10-28

**Authors:** Matvei Khoroshkin, Andrey Buyan, Martin Dodel, Albertas Navickas, Johnny Yu, Fathima Trejo, Anthony Doty, Rithvik Baratam, Shaopu Zhou, Sean B. Lee, Tanvi Joshi, Kristle Garcia, Benedict Choi, Sohit Miglani, Vishvak Subramanyam, Hailey Modi, Christopher Carpenter, Daniel Markett, M. Ryan Corces, Faraz K. Mardakheh, Ivan V. Kulakovskiy, Hani Goodarzi

**Affiliations:** 1 Department of Biochemistry and Biophysics, University of California, San Francisco, San Francisco, CA USA; 2 Department of Urology, University of California, San Francisco, San Francisco, CA USA; 3 Helen Diller Family Comprehensive Cancer Center, University of California, San Francisco, San Francisco, CA USA; 4 Bakar Computational Health Sciences Institute, University of California, San Francisco, San Francisco, CA USA; 5 Institute of Protein Research, Russian Academy of Sciences, Pushchino, Russia; 6 Centre for Cancer Cell and Molecular Biology, Barts Cancer Institute, Queen Mary University of London, London, UK; 7 Department of Biochemistry, University of Oxford, South Parks Road, Oxford, UK; 8 College of Arts and Sciences, University of San Francisco, San Francisco, CA USA; 9 Arc Institute, Palo Alto, CA USA; 10 Gladstone Institute of Neurological Disease, San Francisco, CA USA; 11 Gladstone Institute of Data Science and Biotechnology, San Francisco, CA USA; 12 Department of Neurology, University of California San Francisco, San Francisco, CA USA; 13 Vavilov Institute of General Genetics, Russian Academy of Sciences, Moscow, Russia; 14 Present Address: Institut Curie, UMR3348 CNRS, U1278 Inserm, Orsay France

**Keywords:** Gene regulatory networks, Regulatory networks, RNA metabolism, Functional genomics, Data integration

Correction to: *Nature Communications* 10.1038/s41467-024-52215-7, published online 9 September 2024

In this article, the affiliation ‘Arc Institute, Palo Alto, CA’ was missing for Shaopu Zhou and Hani Goodarzi. The original article has not been corrected.

