# Supplementary material for: Systematic identification of post-transcriptional regulatory modules
Source: Nat Commun. 2024 Sep 9;15:7872. doi: 10.1038/s41467-024-52215-7 (PMC11385195; doi:10.1038/s41467-024-52215-7)
Supplement: Supplementary file 1 — Supplementary Information [file 41467_2024_52215_MOESM1_ESM.pdf]

The PDF file includes:

Figure S1. Multi-modal integration of RBP interaction data
Figure S2. RBPs interaction heatmap inferred from individual data modalities
Figure S3. RBPs integrated distances assessment
Figure S4. The identified RBPs interactions correspond to common functionalities
Figure S5. Estimating cell transcriptional response to RBP depletion with Perturb-seq
Figure S6. BioID2-mediated proximity protein labeling
Figure S7. TAF15 and ZC3H11A regulate alternative splicing
Figure S8. TAF15 controls mRNA translation and stability
Figure S9. ZNF800 and QKI control gene expression at transcriptional and post-transcriptional level

Other Supplementary Material for this manuscript includes the following:

Data file S1 (tsv format). Integrated Regulatory Interaction Map: pairwise distances between RNA binding proteins
Data file S2 (tsv format). The pairwise distances between RBPs inferred from STRING-DB
Data file S3 (tsv format). The pairwise distances between RBPs inferred from BioID2 data
Data file S4 (tsv format). The pairwise distances between RBPs inferred from eCLIP data
Data file S5 (tsv format). The pairwise distances between RBPs inferred from Pertub-seq data
Data file S6 (tsv format). BioID2 Proximity Interactome: log fold changes
Data file S7 (tsv format). BioID2 Proximity Interactome: P-values
Data file S8 (tsv format). eCLIP RBP-RNA binding profiles
Data file S9 (tsv format). Pathway enrichment for RNA binding proteins inferred from BioID2 data: GSEA results for GO Biological Process annotations
Data file S10 (tsv format). Pathway enrichment for RNA binding proteins inferred from BioID2 data: GSEA results for GO Molecular Function annotations
Data file S11 (tsv format). Pathway enrichment for RNA binding proteins inferred from BioID2 data: GSEA results for GO Cellular Component annotations
Data file S12 (tsv format). Data availability for the studied RNA binding proteins
Data file S13 (tsv format). RBP-centered regulatory modules annotation.
Data file S14 (tsv format). Comparison to the external PPI databases STRING, OpenCell and hu.MAP
Data file S15 (tsv format). Compositions of RNA regulons for the RBP-centered regulatory modules.

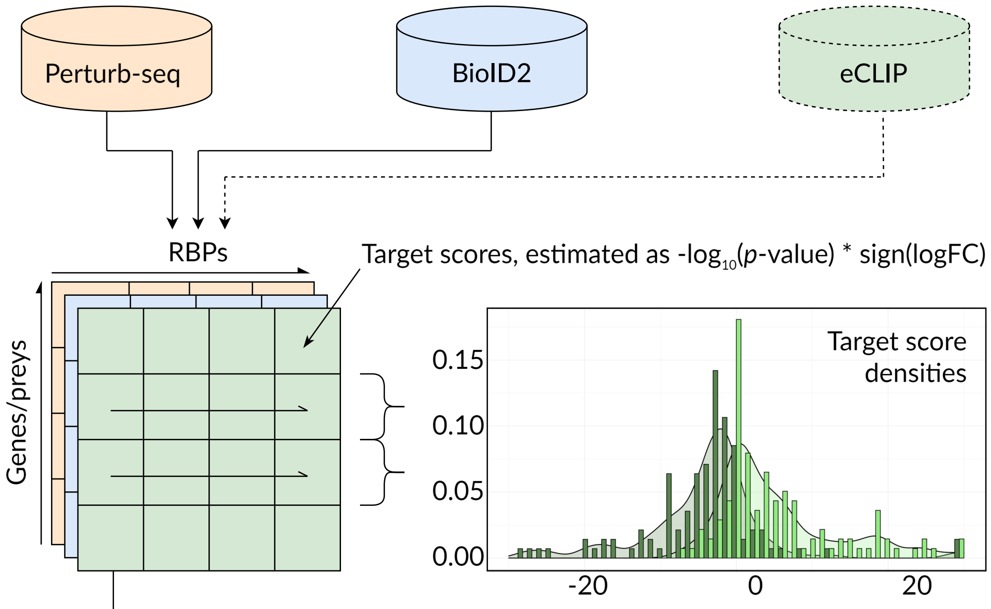

(1) For each gene/prey (for row i):

$$\text{z-score}_i = \frac{\text{target score}_i - \mu_i}{\sigma_i}$$

(2) Calculate inter-RBP cosine distances (for columns i and j):

$$\text{cos dist}_{ij} = 1 - \frac{\sum_{n=1}^{N} \text{z-score}_{ni} \cdot \text{z-score}_{nj}}{\sqrt{\sum_{n=1}^{N} (\text{z-score}_{ni})^2} \cdot \sqrt{\sum_{n=1}^{N} (\text{z-score}_{nj})^2}}$$

RBPs	Perturb-seq	BioID2	eCLIP
RBP1, RBP2	0.8	0.7	0.6
...	...	...	...

Cosine distance

(3) For each RBP pair and dataset (for row i and column j):

$$C = \binom{\text{N}_{RBPs}}{2}, I_{cij} = \begin{cases} 1, \text{ cos dist}_{cj} \leq \text{cos dist}_{ij} \\ 0, \text{ otherwise} \end{cases}$$

$$p\text{-value}_{ij} = \frac{\sum_{c=1}^{C} (I_{cij})}{C}$$

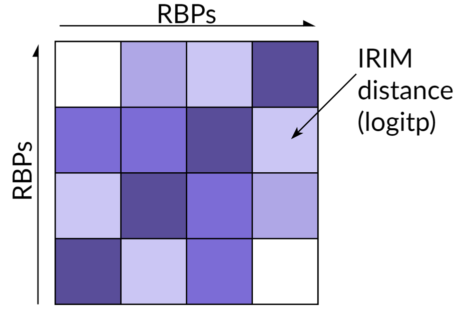

(4) Combine p-values:

$$\text{logitp} = -\frac{\sum_{i=1}^{3} \log\left(\frac{pv_i}{1-pv_i}\right)}{const}$$

Figure S1. Multi-modal integration of RBP interaction data

Top: the three data modalities are shown on top; the datasets generated in this paper are highlighted with solid line, and the data downloaded from publicly available resources is highlighted with dashed line.

Middle: each dataset was preprocessed into a table, where the columns are RBPs and the rows are gene targets (shown in color for individual datasets). Every RBP was represented by a numeric column vector. The gene targets correspond to: for Perturb-Seq - individual genes, for BioID2 - protein binding partners, for eCLIP - mRNA binding targets. Difference between two numeric row vectors is shown on the right in the form of a histogram.

Bottom: the formulas applied at the key steps of the integration procedure are shown. (1): numeric column vectors were normalized by applying z-score transformation. (2): For each dataset, the cosine distances between pairs of individual RBPs were calculated. (3): The resulting distances were then transformed into empirical p-values reflecting assay-specific inter-RBPs distances. (4): Finally, a single interaction score was measured for each RBP pair by combining the p-values from the three assays using logit aggregation.

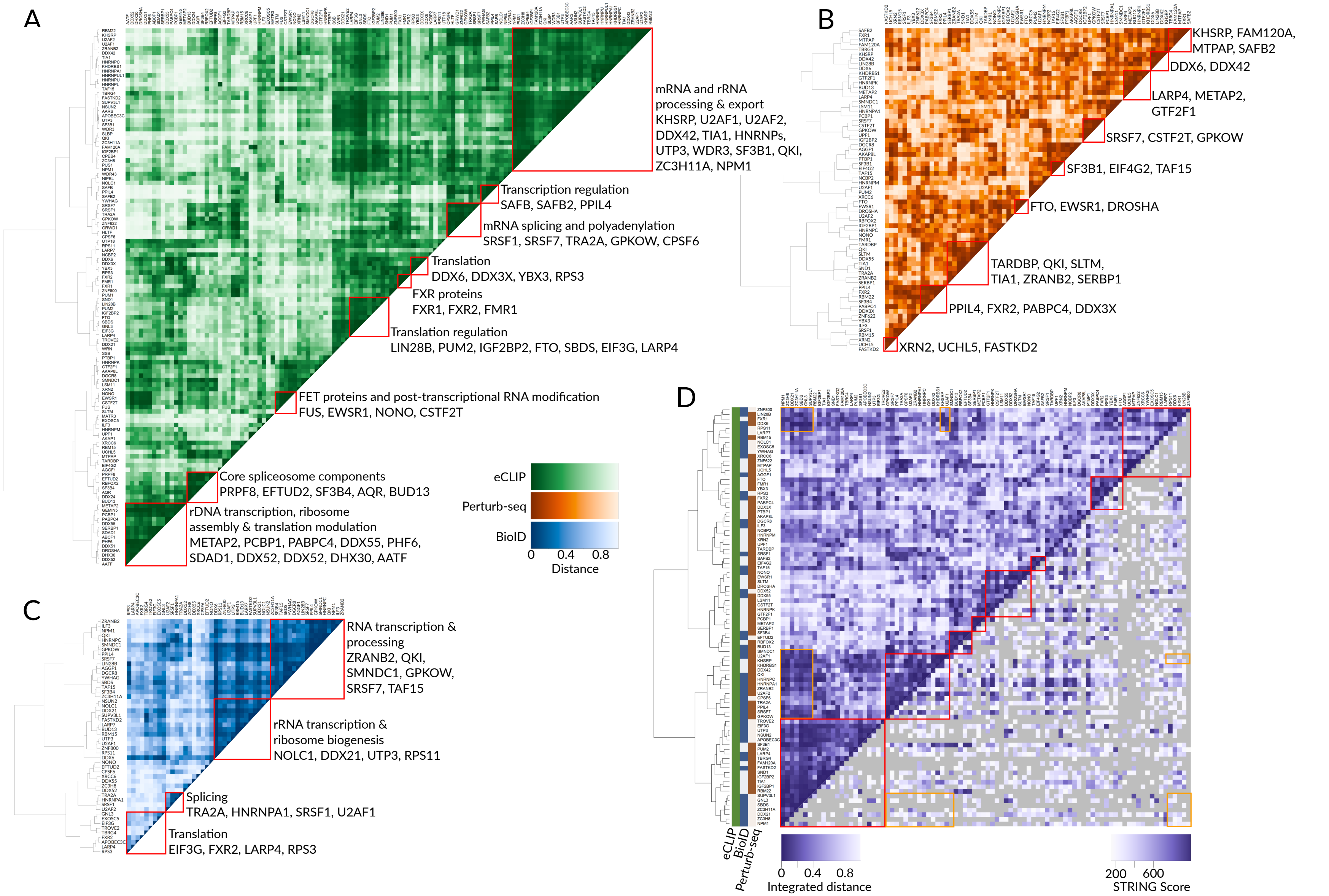
A
mRNA and rRNA processing & export
KHSRP, U2AF1, U2AF2, DDX42, TIA1, HNRNPs, UTP3, WDR3, SF3B1, QKI, ZC3H11A, NPM1
Transcription regulation
SAFB, SAFB2, PPIL4
mRNA splicing and polyadenylation
SRSF1, SRSF7, TRA2A, GPKOW, CPSF6
Translation
DDX6, DDX3X, YBX3, RPS3
FXR proteins
FXR1, FXR2, FMR1
Translation regulation
LIN28B, PUM2, IGF2BP2, FTO, SBDS, EIF3G, LARP4
FET proteins and post-transcriptional RNA modification
FUS, EWSR1, NONO, CSTF2T
Core spliceosome components
PRPF8, EFTUD2, SF3B4, AQR, BUD13
rDNA transcription, ribosome assembly & translation modulation
METAP2, PCBP1, PABPC4, DDX55, PHF6, SDAD1, DDX52, DDX52, DHX30, AATF
B
KHSRP, FAM120A, MTPAP, SAFB2
DDX6, DDX42
LARP4, METAP2, GTF2F1
SRSF7, CSTF2T, GPKOW
SF3B1, EIF4G2, TAF15
FTO, EWSR1, DROSHA
TARDBP, QKI, SLTM, TIA1, ZRANB2, SERBP1
PPIL4, FXR2, PABPC4, DDX3X
XRN2, UCHL5, FASTKD2
eCLIP
Perturb-seq
BioID
0 0.4 0.8
Distance
C
RNA transcription & processing
ZRANB2, QKI, SMNDC1, GPKOW, SRSF7, TAF15
rRNA transcription & ribosome biogenesis
NOLC1, DDX21, UTP3, RPS11
Splicing
TRA2A, HNRNPA1, SRSF1, U2AF1
Translation
EIF3G, FXR2, LARP4, RPS3
D
eCLIP
BioID
Perturb-seq
0 0.4 0.8
Integrated distance
200 600
STRING Score

Figure S2. RBPs interaction heatmap inferred from individual data modalities

(A) A heatmap showing the pairwise distances between RBPs as informed by eCLIP data. Each row and column represents a given RBP; the hierarchical clustering dendrogram of RBPs is shown on the left. Known regulatory modules, consisting of previously annotated functional interactions, are marked with red borders and labeled.

(B) A heatmap showing the pairwise distances between RBPs based on the Perturb-seq data. Annotations as in (A).

(C) A heatmap showing the pairwise distances between RBPs based on the BioID2 dataset. Annotations as in (A).

(D) Upper triangle: the heatmap of IRIM as in Fig. 2A. Lower triangle: the heatmap showing the STRING interaction confidence scores (multiplied by 1000) between RBPs[1]. The location of RBPs within the heatmap is a symmetrical reflection of the upper triangle. The pairs of RBPs where the interaction score could not be calculated are shown in gray. The same regulatory modules as in Fig. 2A are highlighted in red and yellow.

Data used to generate this Figure are available in the Source Data File.

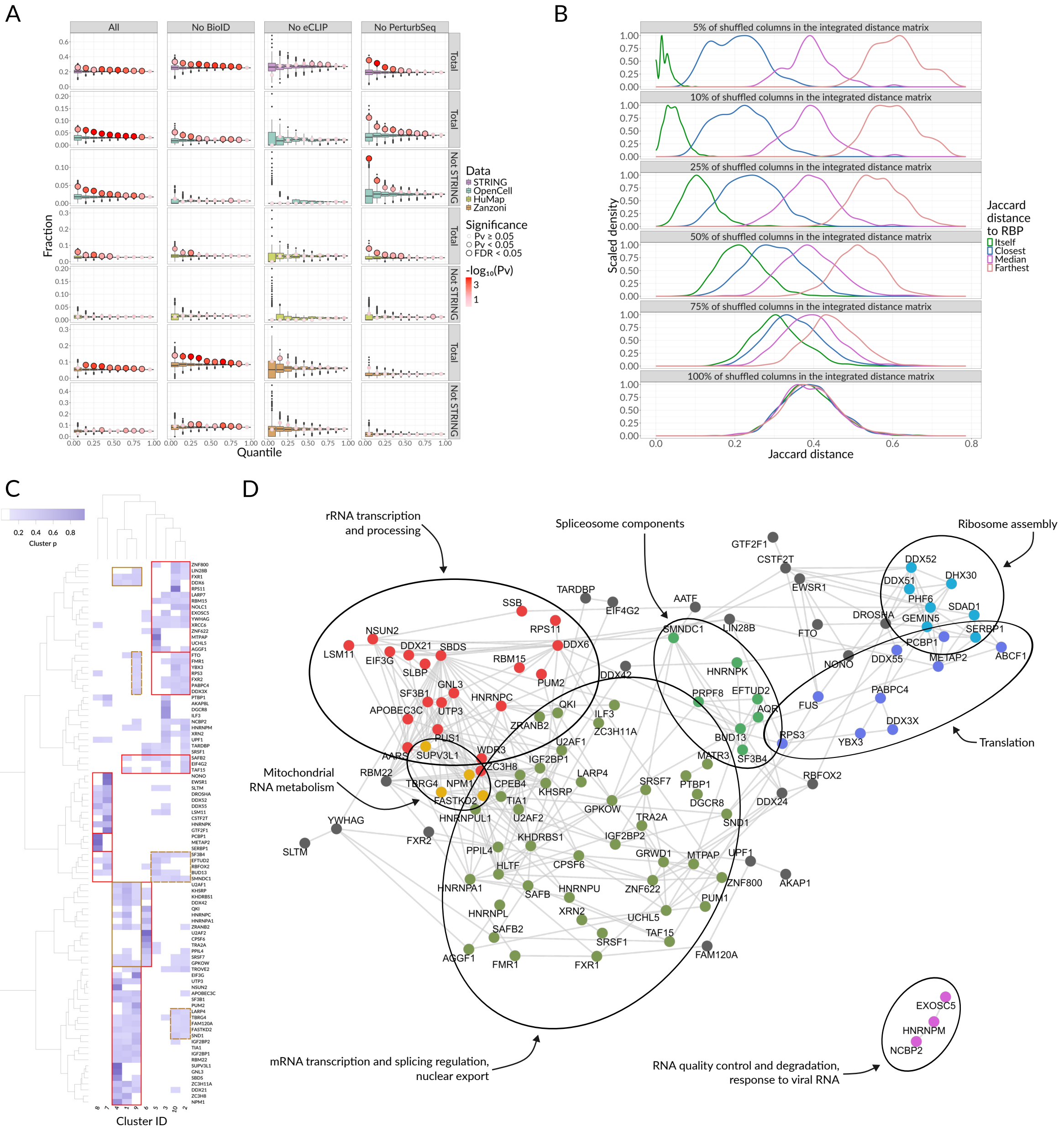
A
All
No BioID
No eCLIP
No PerturbSeq
Total
Not STRING
Fraction
Quantile
Data
STRING
OpenCell
HuMap
Zanzoni
Significance
Pv ≥ 0.05
Pv < 0.05
FDR < 0.05
-log10(Pv)
B
5% of shuffled columns in the integrated distance matrix
10% of shuffled columns in the integrated distance matrix
25% of shuffled columns in the integrated distance matrix
50% of shuffled columns in the integrated distance matrix
75% of shuffled columns in the integrated distance matrix
100% of shuffled columns in the integrated distance matrix
Scaled density
Jaccard distance
Jaccard distance to RBP
Itself
Closest
Median
Farthest
C
Cluster p
Cluster ID
D
rRNA transcription and processing
Spliceosome components
Ribosome assembly
Mitochondrial RNA metabolism
Translation
mRNA transcription and splicing regulation, nuclear export
RNA quality control and degradation, response to viral RNA

Figure S3. RBPs integrated distances assessment

(A) Boxplots and dots representing fractions of the interactions confirmed by the external databases among the RBP pairs with the inter-RBP distance lower than the certain quantile. Boxplots: 10^4 random shuffles of the IRIM; dots: the real data. The distances, left-to-right: the integrated distances from Fig. 2A, the distances from eCLIP and Perturb-seq integration, BioID and Perturb-seq integration, and BioID and eCLIP integration (see Methods). The external databases, top-to-bottom: STRING, OpenCell, OpenCell w/o STRING support, hu.MAP, hu.MAP w/o STRING support, Zanzoni et al., Zanzoni w/o STRING support. Color fill denotes the external database used to calculate the fraction, size and line color denote the right-tailed empirical p-value calculated from 10^4 shuffling iterations (as in Fig. 2F) and FDR-corrected for the number of tested quantiles. Box plot bounds and center represent the first, second and third quartiles, while whiskers represent minimum and maximum values in the data excluding outliers which are more than 1.5 interquartile range from lower and upper quartiles and are depicted as dots.

(B) Density plots illustrating the consistency of IRIM. The distributions of the cosine distances shown in the figure were obtained by shuffling 5%, 10%, 25%, 50%, 75% or 100% of the columns in the pairwise integrated distance matrix (10 shuffles, see Methods). Each time we calculated the cosine distance as the 'distance between distance vectors' for the original RBP's distance vector and (1) its modified version from the shuffled matrix (i.e. distance to the original self), (2) the distance vectors to other RBPs (defining closest, median, and the farthest cosine distances). Considering 90 initial RBPs, the procedure resulted in 90*10 = 900 estimates for each group and shuffling percent.

(C) Fuzzy clustering of IRIM. Rows represent RBPs, columns represent clusters. The functionally pleiotropic groups of RBPs are highlighted in yellow. RBP groups from Fig. 2A are highlighted with solid frames, other groups are highlighted with dashed frames. For the clustering, we used the c-means algorithm with the degree of fuzzification set to 1.25, 10 clusters, and Manhattan distance.

(D) Vertices represent individual RBPs; edges depict the pairs of RBPs with an integrated distance less than 0.05. The communities of RBPs, related to different groups of RNA processes, are colored and highlighted, similar to Fig. 2.

Data used to generate this Figure are available in the Source Data File.

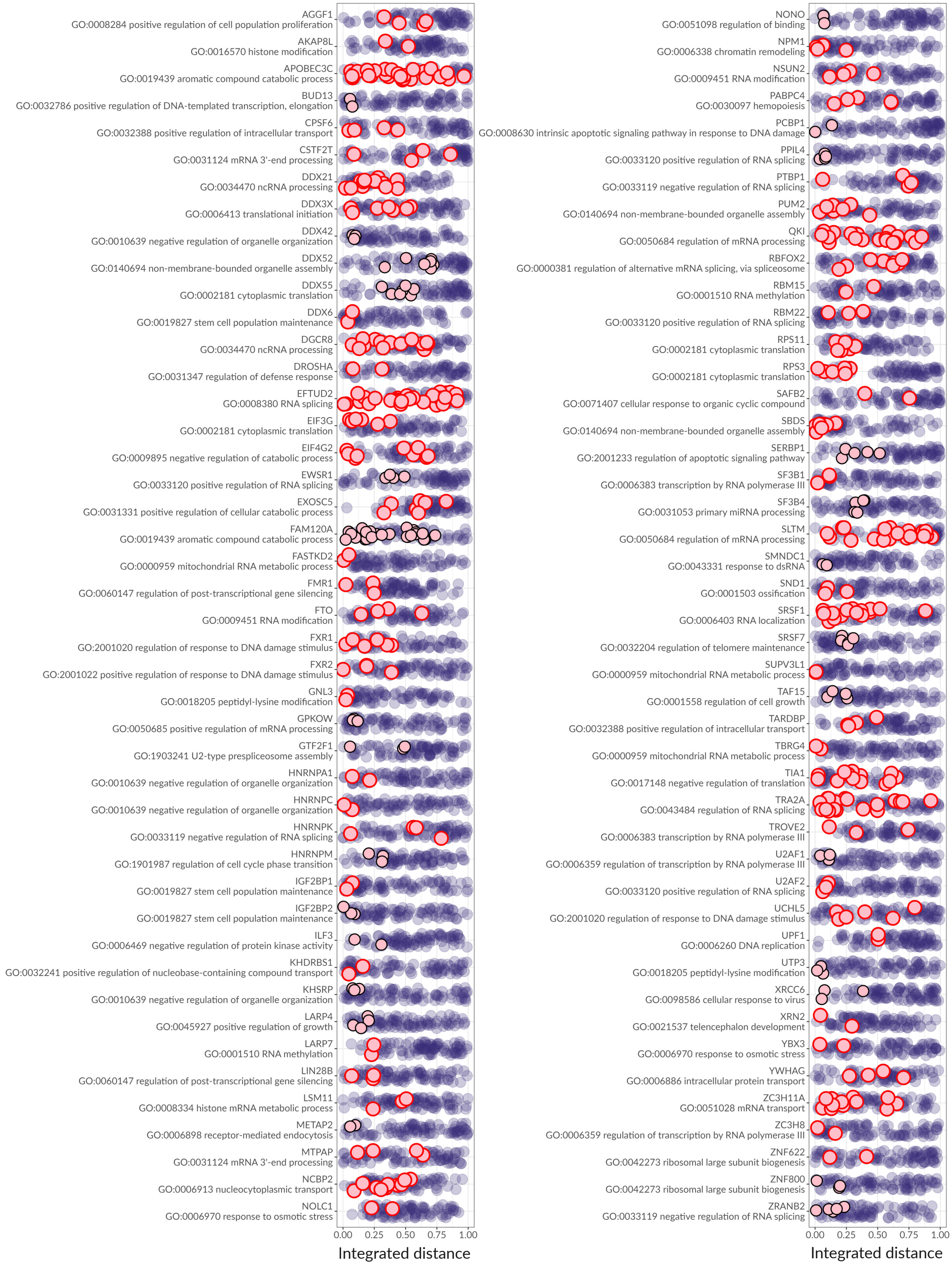
AGGF1
GO:0008284 positive regulation of cell population proliferation
AKAP8L
GO:0016570 histone modification
APOBEC3C
GO:0019439 aromatic compound catabolic process
BUD13
GO:0032786 positive regulation of DNA-templated transcription, elongation
CPSF6
GO:0032388 positive regulation of intracellular transport
CSTF2T
GO:0031124 mRNA 3'-end processing
DDX21
GO:0034470 ncRNA processing
DDX3X
GO:0006413 translational initiation
DDX42
GO:0010639 negative regulation of organelle organization
DDX52
GO:0140694 non-membrane-bounded organelle assembly
DDX55
GO:0002181 cytoplasmic translation
DDX6
GO:0019827 stem cell population maintenance
DGCR8
GO:0034470 ncRNA processing
DROSHA
GO:0031347 regulation of defense response
EFTUD2
GO:0008380 RNA splicing
EIF3G
GO:0002181 cytoplasmic translation
EIF4G2
GO:0009895 negative regulation of catabolic process
EWSR1
GO:0033120 positive regulation of RNA splicing
EXOSC5
GO:0031331 positive regulation of cellular catabolic process
FAM120A
GO:0019439 aromatic compound catabolic process
FASTKD2
GO:0000959 mitochondrial RNA metabolic process
FMR1
GO:0060147 regulation of post-transcriptional gene silencing
FTO
GO:0009451 RNA modification
FXR1
GO:2001020 regulation of response to DNA damage stimulus
FXR2
GO:2001022 positive regulation of response to DNA damage stimulus
GNL3
GO:0018205 peptidyl-lysine modification
GPKOW
GO:0050685 positive regulation of mRNA processing
GTF2F1
GO:1903241 U2-type prespliceosome assembly
HNRNPA1
GO:0010639 negative regulation of organelle organization
HNRNPC
GO:0010639 negative regulation of organelle organization
HNRNPK
GO:0033119 negative regulation of RNA splicing
HNRNPM
GO:1901987 regulation of cell cycle phase transition
IGF2BP1
GO:0019827 stem cell population maintenance
IGF2BP2
GO:0019827 stem cell population maintenance
ILF3
GO:0006469 negative regulation of protein kinase activity
KHDRBS1
GO:0032241 positive regulation of nucleobase-containing compound transport
KHSRP
GO:0010639 negative regulation of organelle organization
LARP4
GO:0045927 positive regulation of growth
LARP7
GO:0001510 RNA methylation
LIN28B
GO:0060147 regulation of post-transcriptional gene silencing
LSM11
GO:0008334 histone mRNA metabolic process
METAP2
GO:0006898 receptor-mediated endocytosis
MTPAP
GO:0031124 mRNA 3'-end processing
NCBP2
GO:0006913 nucleocytoplasmic transport
NOLC1
GO:0006970 response to osmotic stress
0.00
0.25
0.50
0.75
1.00
Integrated distance
NONO
GO:0051098 regulation of binding
NPM1
GO:0006338 chromatin remodeling
NSUN2
GO:0009451 RNA modification
PABPC4
GO:0030097 hemopoiesis
PCBP1
GO:0008630 intrinsic apoptotic signaling pathway in response to DNA damage
PPIL4
GO:0033120 positive regulation of RNA splicing
PTBP1
GO:0033119 negative regulation of RNA splicing
PUM2
GO:0140694 non-membrane-bounded organelle assembly
QKI
GO:0050684 regulation of mRNA processing
RBFOX2
GO:0000381 regulation of alternative mRNA splicing, via spliceosome
RBM15
GO:0001510 RNA methylation
RBM22
GO:0033120 positive regulation of RNA splicing
RPS11
GO:0002181 cytoplasmic translation
RPS3
GO:0002181 cytoplasmic translation
SAFB2
GO:0071407 cellular response to organic cyclic compound
SBDS
GO:0140694 non-membrane-bounded organelle assembly
SERBP1
GO:2001233 regulation of apoptotic signaling pathway
SF3B1
GO:0006383 transcription by RNA polymerase III
SF3B4
GO:0031053 primary miRNA processing
SLTM
GO:0050684 regulation of mRNA processing
SMNDC1
GO:0043331 response to dsRNA
SND1
GO:0001503 ossification
SRSF1
GO:0006403 RNA localization
SRSF7
GO:0032204 regulation of telomere maintenance
SUPV3L1
GO:0000959 mitochondrial RNA metabolic process
TAF15
GO:0001558 regulation of cell growth
TARDBP
GO:0032388 positive regulation of intracellular transport
TBRG4
GO:0000959 mitochondrial RNA metabolic process
TIA1
GO:0017148 negative regulation of translation
TRA2A
GO:0043484 regulation of RNA splicing
TROVE2
GO:0006383 transcription by RNA polymerase III
U2AF1
GO:0006359 regulation of transcription by RNA polymerase III
U2AF2
GO:0033120 positive regulation of RNA splicing
UCHL5
GO:2001020 regulation of response to DNA damage stimulus
UPF1
GO:0006260 DNA replication
UTP3
GO:0018205 peptidyl-lysine modification
XRCC6
GO:0098586 cellular response to virus
XRN2
GO:0021537 telencephalon development
YBX3
GO:0006970 response to osmotic stress
YWHAG
GO:0006886 intracellular protein transport
ZC3H11A
GO:0051028 mRNA transport
ZC3H8
GO:0006359 regulation of transcription by RNA polymerase III
ZNF622
GO:0042273 ribosomal large subunit biogenesis
ZNF800
GO:0042273 ribosomal large subunit biogenesis
ZRANB2
GO:0033119 negative regulation of RNA splicing
0.00
0.25
0.50
0.75
1.00
Integrated distance

Figure S4. The identified RBPs interactions correspond to common functionalities

Swarm Plots for RBP Partners of the 90 RBPs. Each swarm plot represents the ordering of neighboring RBPs for a query RBP. Each point represents an individual RBP. The points are organized by the integrated distance from the specified RBP to the query RBP. Points highlighted in red share the query GO term annotation. The red-circled red dots are a part of the GO term that also includes the query RBP (63 out of 87 RBPs annotated in GO, 72%, 3 RBPs are unannotated); the black-circled red dots represent the GO terms that do not include the query RBP (28% of the annotated RBPs). The query RBP and the query GO term are shown on the left. The common functions of RBPs with their closest neighbors were tested by considering the GO terms that are enriched (NES score < -0.5, focusing on lower distance values) among the close neighbors of each query RBP. These terms were then intersected with the GO terms containing the query protein.

Data used to generate this Figure are available in the Source Data File.

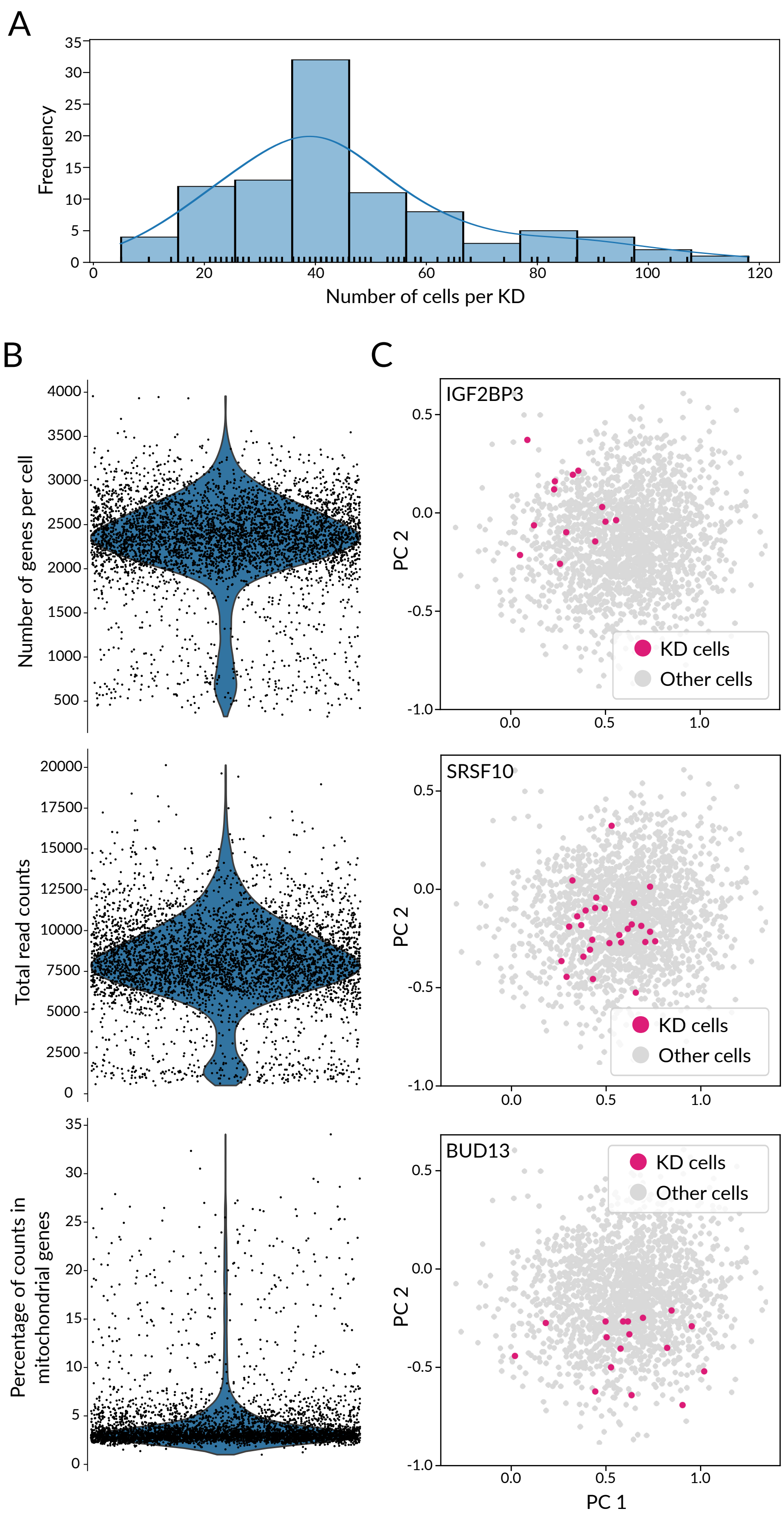
A
Frequency
Number of cells per KD
B
Number of genes per cell
Total read counts
Percentage of counts in mitochondrial genes
C
IGF2BP3
SRSF10
BUD13
KD cells
Other cells
PC 2
PC 1

Figure S5. Estimating cell transcriptional response to RBP depletion with Perturb-seq

(A) A histogram and a Kernel Distribution Estimation Plot showing the number of single cell transcriptomes sequenced for each individual RBP knockdown for the Perturb-seq dataset.

(B) Violin plots showing the number of genes sequenced, number of total reads, and percentage of reads mapping to mitochondrial genes for the single cell transcriptomes sequenced. The cells whose corresponding sgRNA was successfully identified are shown.

(C) PCA projections of the single cell transcriptomes. The cells carrying knock downs of a target RBP are highlighted in red. The knockdown cells are highlighted for example proteins IGF2BP3 (top), SRSF10 (middle) and BUD13 (bottom).

Data used to generate this Figure are available in the Source Data File.

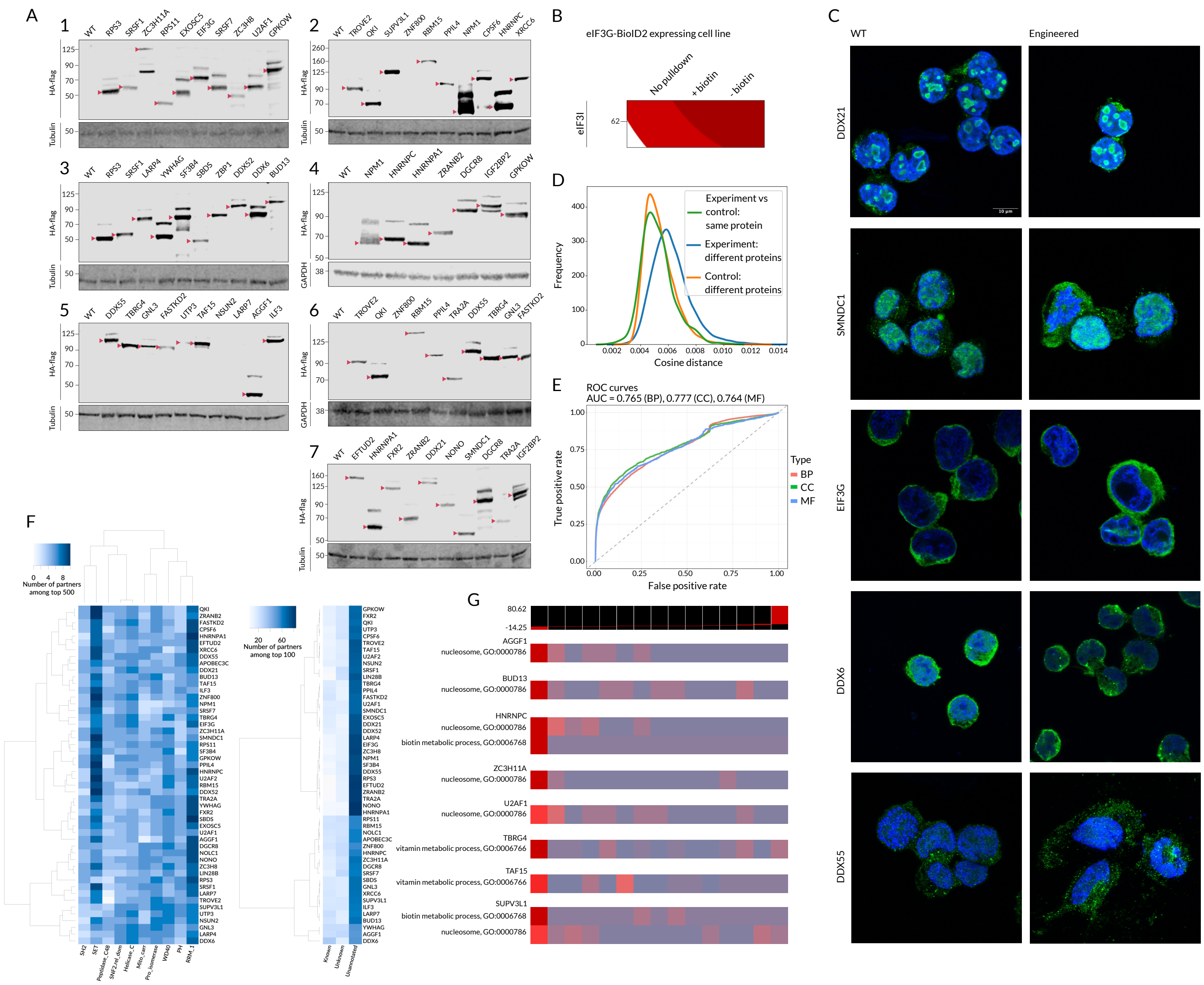

Figure S6. BioID2-mediated proximity protein labeling

(A) Western blot analysis of cell lysates collected from each of 50 RBP-BioID2-expressing cell lines. HA antibody along with either tubulin or GAPDH antibodies for endogenous controls were used. Full blots are shown, the lanes are labeled by the fusion protein expressed; the bands corresponding to the correct protein sizes are highlighted by red arrows. Notably, the presence of multiple bands in Western blots is expected due to recognized post-translational modifications and self-biotinylation of RBPs ([2], Suppl. Fig. 1A).

(B) Western blot analysis of eIF3I in the lysate collected from eIF3G-BioID2-expressing cell line. Input lysate, streptavidin pulldown sample, and negative control pulldown (without the addition of biotin) are shown.

(C) Representative images of immunofluorescence assay results obtained for 5 RBPs WT (left) or fused with BioID2 (right). Blue corresponds to DAPI, green corresponds to RBP antibodies staining.

(D) Kernel Distribution Estimation Plot of pairwise cosine distances between proteomic profiles of all the individual samples. The pairwise distances were grouped into categories based on two features: (1) if the samples are “experiments” (+ biotin) or “control” (- biotin), (2) if the samples come from the same cell line or two different cell lines. The distributions of pairwise distances within 3 representative categories are shown in color.

(E) The ROC curves for predictors of gene ontology (GO) annotations from RBP protein neighborhoods. BioID2-based proximity labeling data was used to predict GO annotations of RBPs as described in Methods. Then, the known GO annotations assigned to a given RBP were used to estimate the specificity and sensitivity of the classifier. The curves corresponding to 3 types of GO annotations are shown in color: BP, MF, and CC represent Biological Process, Molecular Function, and Cellular Component groups of GO, respectively.

(F) Left: heatmap showing the number of proteins representing the most widely present Pfam families among the top 500 neighboring proteins for each query RBP. Right: heatmap showing the fractions of canonical RBPs (those with RNA binding domains), non-canonical RBPs (those with no RNA binding domains that still bind RNA) and unannotated proteins among the top 100 neighboring proteins for each query RBP.

(G) Examples of GO terms significantly depleted in the proximity labeling profiles, as reported by iPAGE. Log fold change values were calculated for biotin-positive versus negative samples. These differences are partitioned into 15 discrete proximity bins. Bins to the left contain proteins that are abundantly found in the negative samples, whereas the ones to the right contain proteins found in biotin-positive samples. In the heat map representation, rows correspond to pathways and columns to consecutive proximity bins. Red entries indicate the enrichment of pathway genes in a given proximity bin. Enrichment and depletion are measured using hypergeometric p-values (log-transformed).

Data used to generate this Figure are available in the Source Data File.

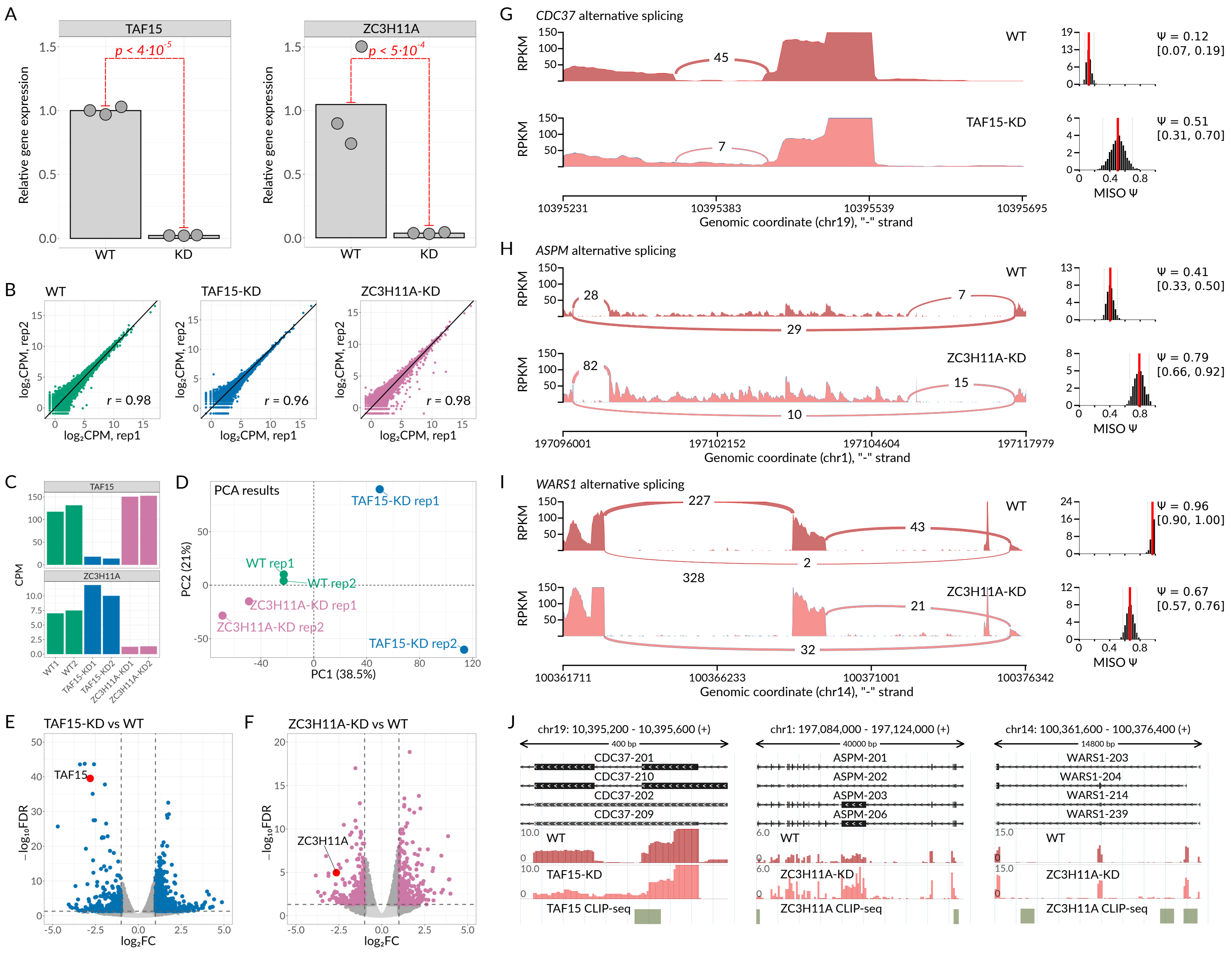

A
TAF15
ZC3H11A
Relative gene expression
p < 4·10-5
p < 5·10-4
WT
KD
B
WT
TAF15-KD
ZC3H11A-KD
log2CPM, rep1
log2CPM, rep2
r = 0.98
r = 0.96
C
CPM
WT1
WT2
TAF15-KD1
TAF15-KD2
ZC3H11A-KD1
ZC3H11A-KD2
D
PCA results
TAF15-KD rep1
WT rep1
WT rep2
ZC3H11A-KD rep1
ZC3H11A-KD rep2
TAF15-KD rep2
PC1 (38.5%)
PC2 (21%)
E
TAF15-KD vs WT
F
ZC3H11A-KD vs WT
−log10FDR
log2FC
G
CDC37 alternative splicing
H
ASPM alternative splicing
I
WARS1 alternative splicing
RPKM
MISO Ψ
Ψ = 0.12 [0.07, 0.19]
Ψ = 0.51 [0.31, 0.70]
Ψ = 0.41 [0.33, 0.50]
Ψ = 0.79 [0.66, 0.92]
Ψ = 0.96 [0.90, 1.00]
Ψ = 0.67 [0.57, 0.76]
Genomic coordinate (chr19), "-" strand
Genomic coordinate (chr1), "-" strand
Genomic coordinate (chr14), "-" strand
J
chr19: 10,395,200 - 10,395,600 (+)
chr1: 197,084,000 - 197,124,000 (+)
chr14: 100,361,600 - 100,376,400 (+)
CDC37-201
CDC37-210
CDC37-202
CDC37-209
ASPM-201
ASPM-202
ASPM-203
ASPM-206
WARS1-203
WARS1-204
WARS1-214
WARS1-239
TAF15 CLIP-seq
ZC3H11A CLIP-seq

Figure S7. TAF15 and ZC3H11A regulate alternative splicing

(A) RT-qPCR quantification of relative levels of *TAF15* and *ZC3H11A* transcripts in the respective knockdown cell lines. *P* from one-sided t-test performed on log-transformed expression estimates, $3.764*10^{-5}$ for TAF15-KD and $4.88*10^{-4}$ for ZC3H11A-KD.

(B) Scatter plots showing the correlations between biological replicates log2CPM for RNA-seq experiments.

(C) Relative expression (in CPM) of *TAF15* and *ZC3H11A* in the RNA-seq samples of WT and knockdown cell lines.

(D) PCA analysis of RNA-seq samples for WT, TAF15-KD and ZC3H11A-KD cells. The first two principal components are shown. Analysis was performed using log2CPM values.

(E) Volcano plot showing the changes in gene expression upon TAF15 knockdown. *TAF15* is highlighted in red. The vertical dashed lines show log2FC thresholds of -1 and 1. The horizontal dashed line corresponds to FDR of 0.05. In total, there are 930 genes passing |log2FC| > 1 and FDR < 0.05 (highlighted in blue)

(F) Volcano plot showing the changes in gene expression upon ZC3H11A knockdown with 565 genes passing |log2FC| > 1 and FDR < 0.05 filters (highlighted in pink). The filters are shown as in (D). *ZC3H11A* is highlighted in red.

(G) Sashimi plot illustrating the changes in intron retention event usage in *CDC37* transcript upon TAF15 knockdown.

(H) Sashimi plot illustrating the changes in skipped exon usage in *ASPM* transcript upon ZC3H11A knockdown.

(I) Sashimi plot illustrating the changes in skipped exon usage in *WARS1* transcript upon ZC3H11A knockdown.

(J) Genomic views of the *CDC37* retained intron (left), *ASPM* skipped exon (middle), and *WARS1* skipped exon (right). Below, the RNA-seq profiles from WT, TAF15-KD and ZC3H11A-KD cells are shown. Y axis: counts per million (CPM). TAF15 and ZC3H11A CLIP-seq peaks are shown at the bottom.

Data used to generate this Figure are available in the Source Data File.

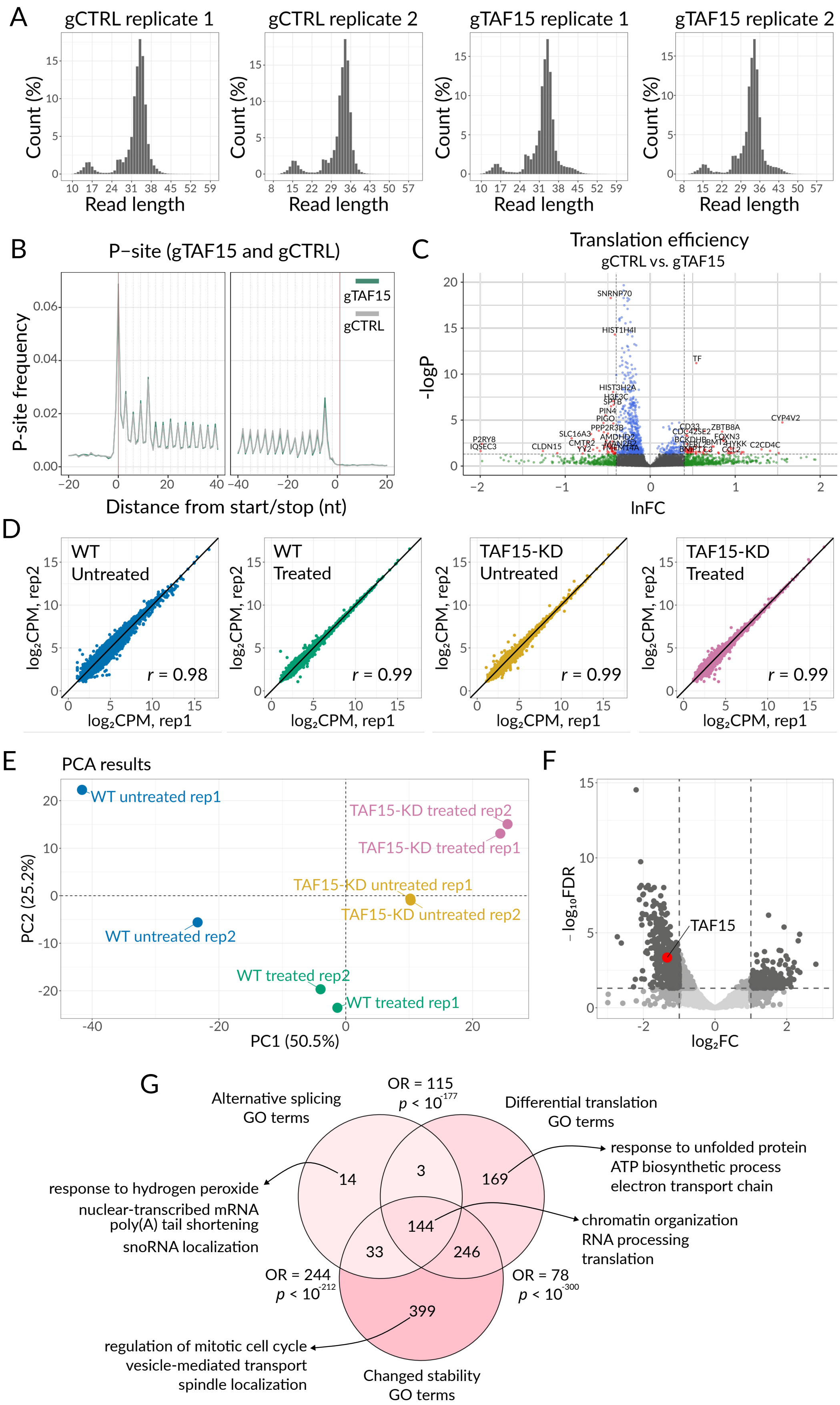

A
gCTRL replicate 1
gCTRL replicate 2
gTAF15 replicate 1
gTAF15 replicate 2
Count (%)
Read length
B
P−site (gTAF15 and gCTRL)
P-site frequency
gTAF15
gCTRL
Distance from start/stop (nt)
C
Translation efficiency
gCTRL vs. gTAF15
-logP
lnFC
SNRNP70
HIST1H4I
TF
HIST1H2A
CYP4V2
D
WT
Untreated
WT
Treated
TAF15-KD
Untreated
TAF15-KD
Treated
log2CPM, rep2
log2CPM, rep1
r = 0.98
r = 0.99
r = 0.99
r = 0.99
E
PCA results
WT untreated rep1
TAF15-KD treated rep2
TAF15-KD treated rep1
TAF15-KD untreated rep1
TAF15-KD untreated rep2
WT untreated rep2
WT treated rep2
WT treated rep1
PC2 (25.2%)
PC1 (50.5%)
F
TAF15
−log10FDR
log2FC
G
Alternative splicing
GO terms
OR = 115
p < 10^-177
Differential translation
GO terms
14
3
169
response to hydrogen peroxide
nuclear-transcribed mRNA
poly(A) tail shortening
snoRNA localization
response to unfolded protein
ATP biosynthetic process
electron transport chain
144
chromatin organization
RNA processing
translation
33
246
OR = 244
p < 10^-212
OR = 78
p < 10^-300
399
regulation of mitotic cell cycle
vesicle-mediated transport
spindle localization
Changed stability
GO terms

Figure S8. TAF15 controls mRNA translation and stability

(A) Distribution of RPFs, aligned on an inferred ribosome P-site, on a metagene, centered around the translation start (left) or stop (right) site, for Ribo-seq of TAF15-KD and WT cells.

(B) Length distribution of ribosome protected footprints (RPFs) as determined by Ribo-seq.

(C) Volcano plot illustrating the changes in ribosome occupancy in TAF15-KD compared to control K562 cells, as determined by ribosome profiling analysis. The data points are colored according to thresholds in effect size (lnFC ± ln1.5) and significance (p-adjusted < 0.05, t-test). The genes passing both significance filters are labeled.

(D) Scatter plots showing the correlations between biological replicates log2CPM for RNA-seq analysis of α-amanitin treated and untreated WT and TAF15-KD cells. The different treatments and cell lines are shown in color.

(E) PCA analysis of RNA-seq samples for α-amanitin treated and untreated WT and TAF15-KD cells. The first two principal components are shown. Analysis was performed using log2CPM values.

(F) Volcano plot showing genes differential stability upon α-amanitin treatment, in TAF15-KD compared to control K562 cells. *TAF15* is highlighted red. The vertical dashed lines show log2FC thresholds of -1 and 1. The horizontal dashed line corresponds to FDR of 0.05. In total, there are 1015 genes passing |log2FC| > 1 and FDR < 0.05 (highlighted in dark gray).

(G) Venn diagram of TAF15 RNA regulons. Shown are the numbers of significantly enriched GO terms (FDR < 0.05) for genes that exhibit significant changes in splicing, stability, or translation upon TAF15 knockdown, as captured by RNA-seq, Ribo-seq, and RNA-seq with α-amanitin, respectively. Manually selected representative GO terms are shown for each part of the diagram. Results of one-sided fisher's exact test for each pairwise intersection are shown next to the corresponding area (p-value = $2.93*10^{-178}$ for differential translation and splicing intersection, $1.78*10^{-213}$ for differential stability and splicing, and less than 10^{-300} for differential translation and stability).

Data used to generate this Figure are available in the Source Data File.

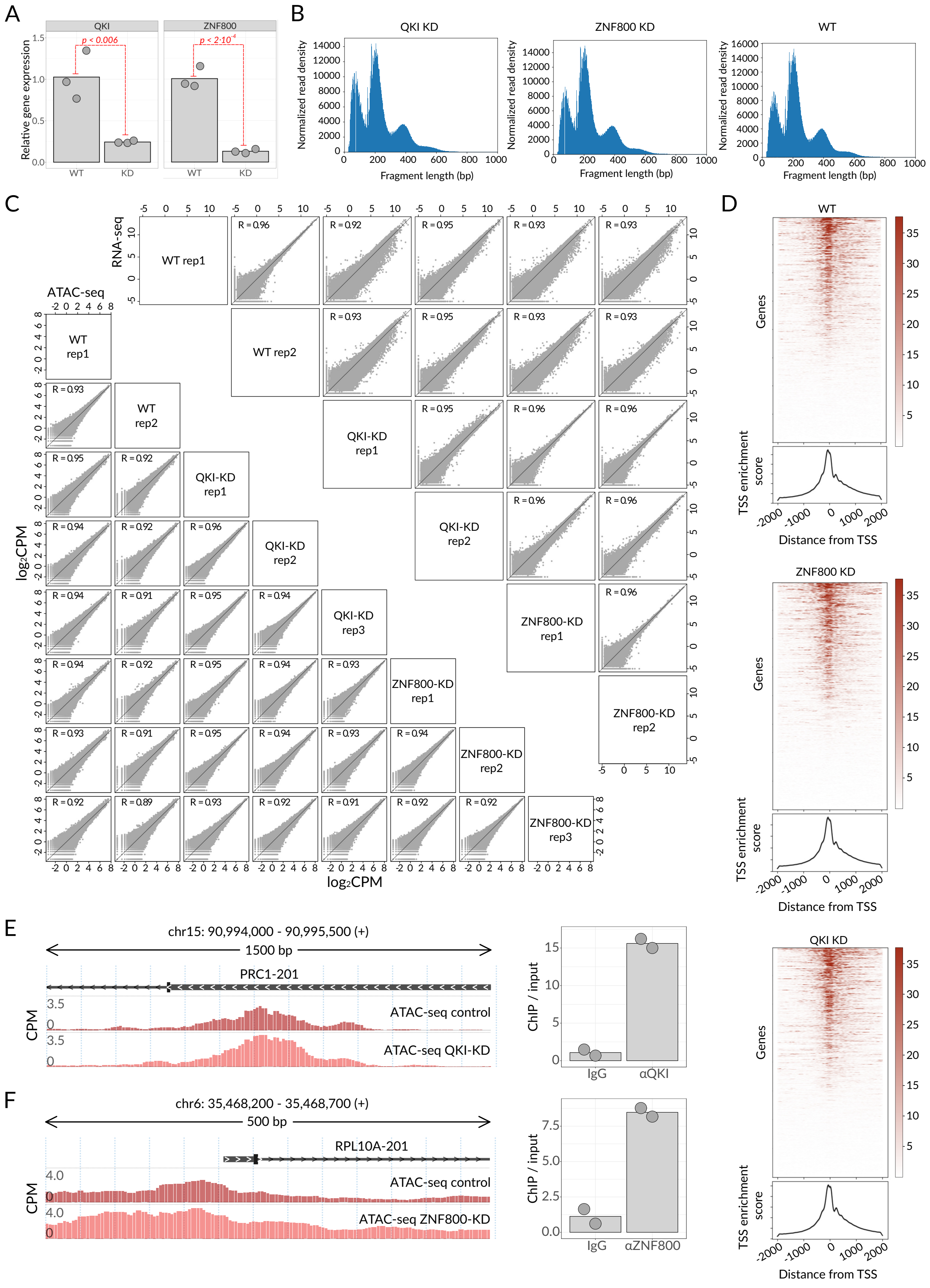
A
QKI
ZNF800
Relative gene expression
p < 0.006
p < 2·10⁻⁴
WT
KD
B
QKI KD
ZNF800 KD
WT
Normalized read density
Fragment length (bp)
C
RNA-seq
ATAC-seq
log₂CPM
WT rep1
WT rep2
QKI-KD rep1
QKI-KD rep2
QKI-KD rep3
ZNF800-KD rep1
ZNF800-KD rep2
ZNF800-KD rep3
D
WT
ZNF800 KD
QKI KD
Genes
TSS enrichment score
Distance from TSS
E
chr15: 90,994,000 - 90,995,500 (+)
1500 bp
PRC1-201
CPM
ATAC-seq control
ATAC-seq QKI-KD
ChIP / input
IgG
αQKI
F
chr6: 35,468,200 - 35,468,700 (+)
500 bp
RPL10A-201
ATAC-seq control
ATAC-seq ZNF800-KD
αZNF800

Figure S9. ZNF800 and QKI control gene expression at transcriptional and post-transcriptional level

(A) RT-qPCR quantification of relative levels of *QKI* and *ZNF800* transcripts in the respective knockdown cell lines. *P* from one-sided t-test performed on log-transformed expression estimates, 0.0052 for QKI-KD and $1.18*10^{-4}$ for ZNF800-KD.

(B) Histograms showing the fragment length distributions for ATAC-seq experiments for QKI-KD cells (left), ZNF800-KD cells (middle), and WT cells (right). A single exemplar replicate is shown per experiment.

(C) Scatter plots showing the correlations between biological replicates log2CPM for RNA-seq experiments (top) and ATAC-seq experiments (bottom).

(D) Enrichment of ATAC-seq reads near transcription start sites (TSS) is shown with heatmaps and Kernel Distribution Estimation (KDE) plots for WT cells (top), ZNF800-KD cells (middle), and QKI-KD cells (bottom). Heatmaps: rows correspond to genes; columns correspond to genomic positions relative to TSSs. Cell values show the number of ATAC-seq reads aligned to a given region. KDE plots show TSS enrichment scores across genes. A single exemplar replicate is shown per experiment.

(E) Left: genomic view of *PRC1* promoter region. ATAC-seq profiles of WT cells and QKI-KD cells are shown. Right: binding of QKI to the *PRC1* promoter region as measured by ChIP-qPCR in K562 cells.

(F) Left: genomic view of *RPL10A* promoter region. ATAC-seq profiles of WT cells and ZNF800-KD cells are shown. Right: binding of ZNF800 to the *RPL10A* promoter region as measured by ChIP-qPCR in K562 cells.

Data used to generate this Figure are available in the Source Data File.

Supplementary References

1. Szklarczyk, D. *et al.* STRING v11: protein–protein association networks with increased coverage, supporting functional discovery in genome-wide experimental datasets. *Nucleic Acids Res.* 47, D607–D613 (2018).

2. Youn, J.-Y. *et al.* High-Density Proximity Mapping Reveals the Subcellular Organization of mRNA-Associated Granules and Bodies. *Mol. Cell* 69, 517–532.e11 (2018).
