# Supplementary material for: Systematic identification of post-transcriptional regulatory modules
Source: Nat Commun. 2024 Sep 9;15:7872. doi: 10.1038/s41467-024-52215-7 (PMC11385195; doi:10.1038/s41467-024-52215-7)
Supplement: Supplementary file 3 — Description of Additional Supplementary Files [file 41467_2024_52215_MOESM3_ESM.pdf]

Description of Additional Supplementary Files:

Supplementary Data 1: (Microsoft Excel format). Integrated Regulatory Interaction Map: pairwise distances between RNA binding proteins

Supplementary Data 2: (Microsoft Excel format). The pairwise distances between RBPs inferred from STRING-DB

Supplementary Data 3: (Microsoft Excel format). The pairwise distances between RBPs inferred from BioID2 data

Supplementary Data 4: (Microsoft Excel format). The pairwise distances between RBPs inferred from eCLIP data

Supplementary Data 5: (Microsoft Excel format). The pairwise distances between RBPs inferred from Pertub-seq data

Supplementary Data 6: (Microsoft Excel format). BioID2 Proximity Interactome: log fold changes

Supplementary Data 7: (Microsoft Excel format). BioID2 Proximity Interactome: P-values
Data file S8 (Microsoft Excel format). eCLIP RBP-RNA binding profiles

Supplementary Data 9: (tsv format). Pathway enrichment for RNA binding proteins inferred from BioID2 data: GSEA results for GO Biological Process annotations

Supplementary Data 10: (tsv format). Pathway enrichment for RNA binding proteins inferred from BioID2 data: GSEA results for GO Molecular Function annotations

Supplementary Data 11: (tsv format). Pathway enrichment for RNA binding proteins inferred from BioID2 data: GSEA results for GO Cellular Component annotations

Supplementary Data 12: (Microsoft Excel format). Data availability for the studied RNA binding proteins

Supplementary Data 13: (Microsoft Excel format). RBPcentered regulatory modules annotation.

Supplementary Data 14: (Microsoft Excel format). Comparison to the external PPI databases STRING, OpenCell and hu.MAP

Supplementary Data 15: (Microsoft Excel format). Compositions of RNA regulons for the RBPcentered regulatory modules.
