# Supplementary material for: Systematic identification of post-transcriptional regulatory modules
Source: Nat Commun. 2024 Sep 9;15:7872. doi: 10.1038/s41467-024-52215-7 (PMC11385195; doi:10.1038/s41467-024-52215-7)
Supplement: Supplementary file 19 — Reporting Summary [file 41467_2024_52215_MOESM19_ESM.pdf]

nature portfolio

Corresponding author(s): Hani Goodarzi

Last updated by author(s): 07-24-2024

Reporting Summary

Nature Portfolio wishes to improve the reproducibility of the work that we publish. This form provides structure for consistency and transparency in reporting. For further information on Nature Portfolio policies, see our Editorial Policies and the Editorial Policy Checklist.

Statistics

For all statistical analyses, confirm that the following items are present in the figure legend, table legend, main text, or Methods section.

n/a	Confirmed	
☐	☒	The exact sample size (*n*) for each experimental group/condition, given as a discrete number and unit of measurement
☐	☒	A statement on whether measurements were taken from distinct samples or whether the same sample was measured repeatedly
☐	☒	The statistical test(s) used AND whether they are one- or two-sided<br>*Only common tests should be described solely by name; describe more complex techniques in the Methods section.*
☐	☒	A description of all covariates tested
☐	☒	A description of any assumptions or corrections, such as tests of normality and adjustment for multiple comparisons
☐	☒	A full description of the statistical parameters including central tendency (e.g. means) or other basic estimates (e.g. regression coefficient) AND variation (e.g. standard deviation) or associated estimates of uncertainty (e.g. confidence intervals)
☐	☒	For null hypothesis testing, the test statistic (e.g. F, t, r) with confidence intervals, effect sizes, degrees of freedom and P value noted<br>*Give P values as exact values whenever suitable.*
☒	☐	For Bayesian analysis, information on the choice of priors and Markov chain Monte Carlo settings
☒	☐	For hierarchical and complex designs, identification of the appropriate level for tests and full reporting of outcomes
☐	☒	Estimates of effect sizes (e.g. Cohen's d, Pearson's r), indicating how they were calculated

Our web collection on statistics for biologists contains articles on many of the points above.

Software and code

Policy information about availability of computer code

Data collection	All the sequencing data has been deposited at Gene Expression Omnibus (GSE225809), and are publicly available as of the date of publication. The processed data has been deposited to Zenodo (identifier 11556393). The list of bona-fide BioID protein pairs has been deposited to IMEx (identifier IM-30059). The mass spectrometry proteomics data have been deposited to the ProteomeXchange Consortium via the PRIDE partner repository with the dataset identifier PXD041608.
Data analysis	All of the original code is available at https://github.com/goodarzilab/RBP_modules

For manuscripts utilizing custom algorithms or software that are central to the research but not yet described in published literature, software must be made available to editors and reviewers. We strongly encourage code deposition in a community repository (e.g. GitHub). See the Nature Portfolio guidelines for submitting code & software for further information.

Data

Policy information about availability of data

All manuscripts must include a data availability statement. This statement should provide the following information, where applicable:

- Accession codes, unique identifiers, or web links for publicly available datasets
- A description of any restrictions on data availability
- For clinical datasets or third party data, please ensure that the statement adheres to our policy

All the sequencing data has been deposited at Gene Expression Omnibus (GSE225809), and are publicly available as of the date of publication. The processed data has been deposited to Zenodo (identifier 11556393). The list of bona-fide BioID protein pairs has been deposited to IMEx (identifier IM-30059). The mass

spectrometry proteomics data have been deposited to the ProteomeXchange Consortium via the PRIDE partner repository with the dataset identifier PXD041608.

Human research participants

Policy information about studies involving human research participants and Sex and Gender in Research.

Reporting on sex and gender	*Use the terms sex (biological attribute) and gender (shaped by social and cultural circumstances) carefully in order to avoid confusing both terms. Indicate if findings apply to only one sex or gender; describe whether sex and gender were considered in study design whether sex and/or gender was determined based on self-reporting or assigned and methods used. Provide in the source data disaggregated sex and gender data where this information has been collected, and consent has been obtained for sharing of individual-level data; provide overall numbers in this Reporting Summary. Please state if this information has not been collected. Report sex- and gender-based analyses where performed, justify reasons for lack of sex- and gender-based analysis.*
Population characteristics	*Describe the covariate-relevant population characteristics of the human research participants (e.g. age, genotypic information, past and current diagnosis and treatment categories). If you filled out the behavioural & social sciences study design questions and have nothing to add here, write "See above."*
Recruitment	*Describe how participants were recruited. Outline any potential self-selection bias or other biases that may be present and how these are likely to impact results.*
Ethics oversight	*Identify the organization(s) that approved the study protocol.*

Note that full information on the approval of the study protocol must also be provided in the manuscript.

Field-specific reporting

Please select the one below that is the best fit for your research. If you are not sure, read the appropriate sections before making your selection.

[x] Life sciences [ ] Behavioural & social sciences [ ] Ecological, evolutionary & environmental sciences

For a reference copy of the document with all sections, see nature.com/documents/nr-reporting-summary-flat.pdf

Life sciences study design

All studies must disclose on these points even when the disclosure is negative.

Sample size	Statistical parameters are reported in the figures and figure legends, including the definitions and experimental measures depicted either as bar charts representing mean and dot plots representing exact values or as boxplots representing median, 25th and 75th percentile (boxes) and 5% and 95% confidence intervals (error bars). For BioID-based RBP annotation procedure, statistical significance is indicated by asterisks * if GSEA FDR adjusted P < 0.05. Pairwise comparisons of qPCR results and log-transformed MS intensity ratios were performed using a one-sided t-test (for testing alternative splicing) or Wilcoxon rank sum test (for testing protein levels and mRNA relative stability). Exact p-values are depicted above the corresponding bar charts. For TAF15 mRNA target enrichment analysis, GSEA statistics including p-values and enrichment scores are depicted in the figure. To test the intersection of different TAF15 regulons, p-values were calculated using one-sided Fisher's exact tests with the statistical significance indicated by asterisks *, p-value < 0.05, **, p-value < 10-5. Pairwise comparisons of the QKI and ZNF800 targets expression level and chromatin accessibility were performed using a one-sided Wilcoxon rank sum test with exact p-values depicted above the boxplots.
Data exclusions	No data was excluded from our analyses
Replication	BioID2-pulldown followed by mass spectrometry was performed in 3 replicates<br>Perturb-seq was performed in 3 replicates<br>ATAC-seq was performed in 3 replicates<br>Ribosome profiling was performed in 2 replicates
Randomization	Randomization is not relevant to our study
Blinding	Blinding was not relevant to our study.

Reporting for specific materials, systems and methods

We require information from authors about some types of materials, experimental systems and methods used in many studies. Here, indicate whether each material, system or method listed is relevant to your study. If you are not sure if a list item applies to your research, read the appropriate section before selecting a response.

Materials & experimental systems

n/a	Involved in the study	
	☒	Antibodies
	☒	Eukaryotic cell lines
☒		Palaeontology and archaeology
☒		Animals and other organisms
☒		Clinical data
☒		Dual use research of concern

Methods

n/a	Involved in the study	
☒		ChIP-seq
☒		Flow cytometry
☒		MRI-based neuroimaging

Antibodies

Antibodies used

HA antibody Biolegend CAT# 901533
eIF3I antibody Biolegend CAT# 646701
Beta-tubulin antibody Proteintech CAT# 10094-1-AP
GAPDH antibody Proteintech CAT# 10494-1-AP
TAF15 antibody Thermo CAT# MA3-078
ZC3H11A antibody Abcam CAT# AB241612
EIF3G Antibody Novus Biologicals CAT# NBP1-84872-25ul
DDX6 Antibody Novus Biologicals CAT# NB200-191
FASTKD Antibody Novus Biologicals CAT# NBP2-57917-25ul
DDX21 Antibody Novus Biologicals CAT# NB100-1718
SMNDC1 Antibody Novus Biologicals CAT# NBP2-20424
Alexa Fluor® 594 Donkey Anti-Rabbit IgG Jackson Immunoresearch Labs CAT# 711-585-152

Validation

HA antibody Biolegend CAT# 901533
Tested and reported applications of the 16B12 clone for the relevant formats include: western blot (WB), immunocytochemistry (ICC), immunoprecipitation (IP), and flow cytometry (FC).

eIF3I antibody Biolegend CAT# 646701
Tested for western blotting.

Beta-tubulin antibody Proteintech CAT# 10094-1-AP
Positive WB detected in U-251 cells, mouse kidney tissue, HEK-293 cells, rat kidney tissue, mouse brain tissue, rat brain tissue.

GAPDH antibody Proteintech CAT# 10494-1-AP
Positive WB detected in human placenta tissue, HepG2 cells, HEK-293 cells, HeLa cells, Raji cells, K-562 cells, mouse heart tissue, PC-13 cells, arabidopsis whole plant tissue, corn whole plant tissue, mouse brain tissue, mouse skin tissue, Jurkat cells, NIH/3T3 cells, C6 cells, rat brain tissue, RAW 264.7 cells.

TAF15 antibody Thermo CAT# MA3-078
Tested and reported applications: Western blot, Immunofluorescent analysis, Flow cytometry, immunoprecipitation.

ZC3H11A antibody Abcam CAT# AB241612
Tested applications: WB, ICC/IF, IP, Flow Cyt (Intra).

EIF3G Antibody Novus Biologicals CAT# NBP1-84872-25ul
Tested applications: Immunohistochemistry, Western Blot

DDX6 Antibody Novus Biologicals CAT# NB200-191
Tested applications: Immunohistochemistry, Western Blot

FASTKD Antibody Novus Biologicals CAT# NBP2-57917-25ul
Tested applications: Immunocytochemistry, Immunofluorescence

DDX21 Antibody Novus Biologicals CAT# NB100-1718
Tested applications: Chromatin Immunoprecipitation, Flow Cytometry, Immunocytochemistry, Western Blot

SMNDC1 Antibody Novus Biologicals CAT# NBP2-20424
Tested applications: Immunocytochemistry, Immunofluorescence, Western Blot

Alexa Fluor® 594 Donkey Anti-Rabbit IgG Jackson Immunoresearch Labs CAT# 711-585-152
Tested applications: Immunocytochemistry, Immunofluorescence

Eukaryotic cell lines

Policy information about cell lines and Sex and Gender in Research

Cell line source(s)	K562 cells were purchased from ATCC
Authentication	K562 cells were authentificated
Mycoplasma contamination	Cell lines were tested regularly for mycoplasma contamination.
Commonly misidentified lines (See ICLAC register)	No commonly misidentified samples were used in this study
