# Supplementary figures and images for: Systematic identification of post-transcriptional regulatory modules
Source: Nat Commun. 2024 Sep 9;15:7872. doi: 10.1038/s41467-024-52215-7 (PMC11385195; doi:10.1038/s41467-024-52215-7)

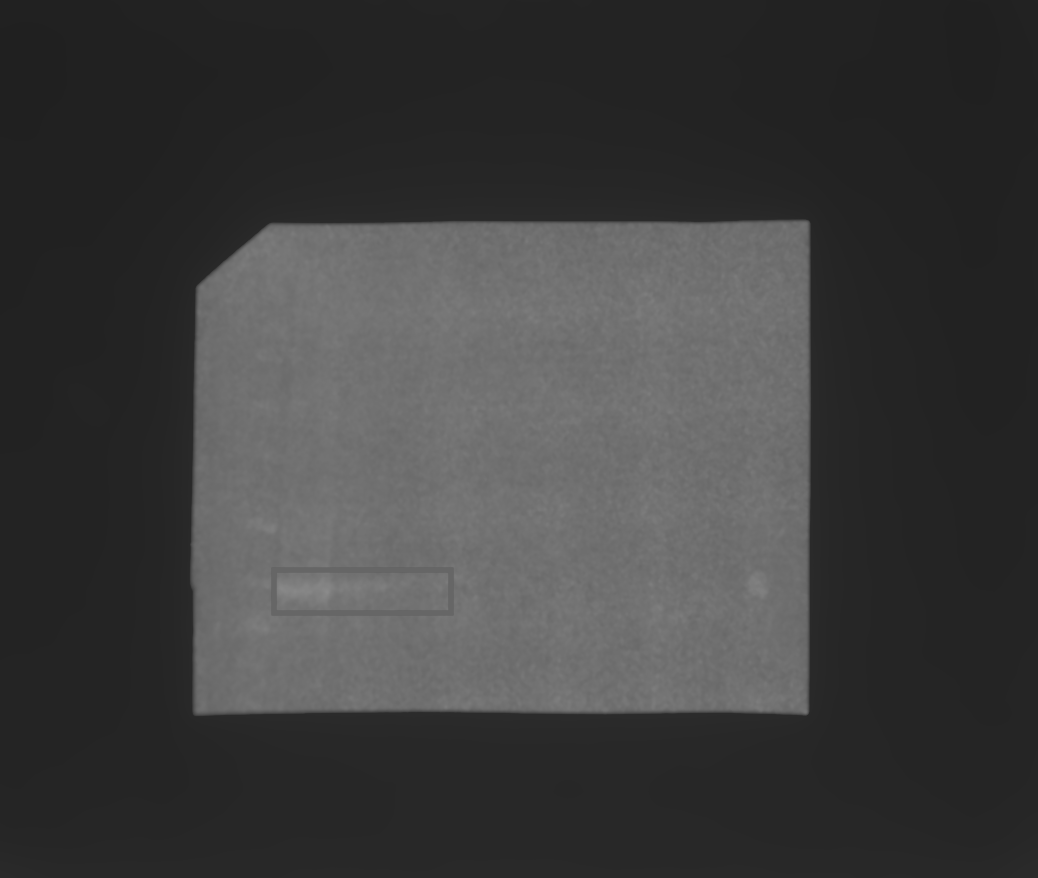

Supplement: Supplementary file 20 — Source Data [file 41467_2024_52215_MOESM20_ESM.zip › Source_data/FigS6B_western_tubulin.TIF]

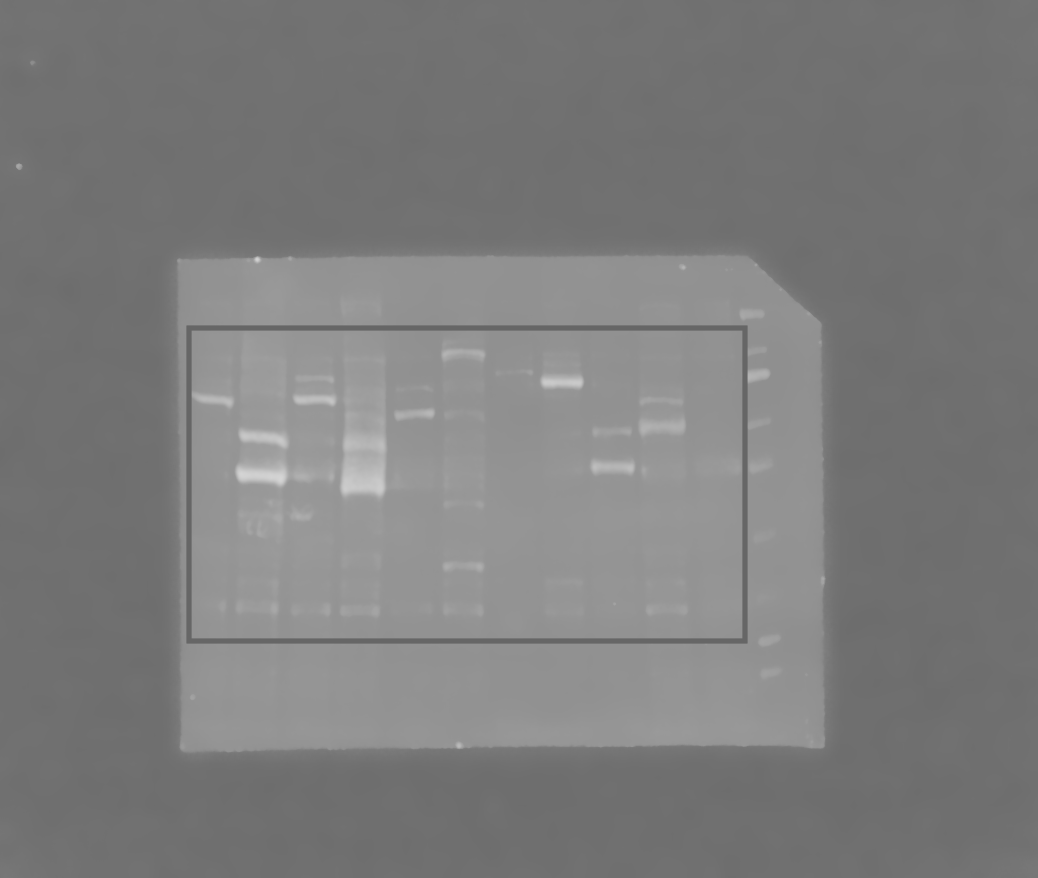

Supplement: Supplementary file 20 — Source Data [file 41467_2024_52215_MOESM20_ESM.zip › Source_data/FigS6A_western_2_HA.TIF]

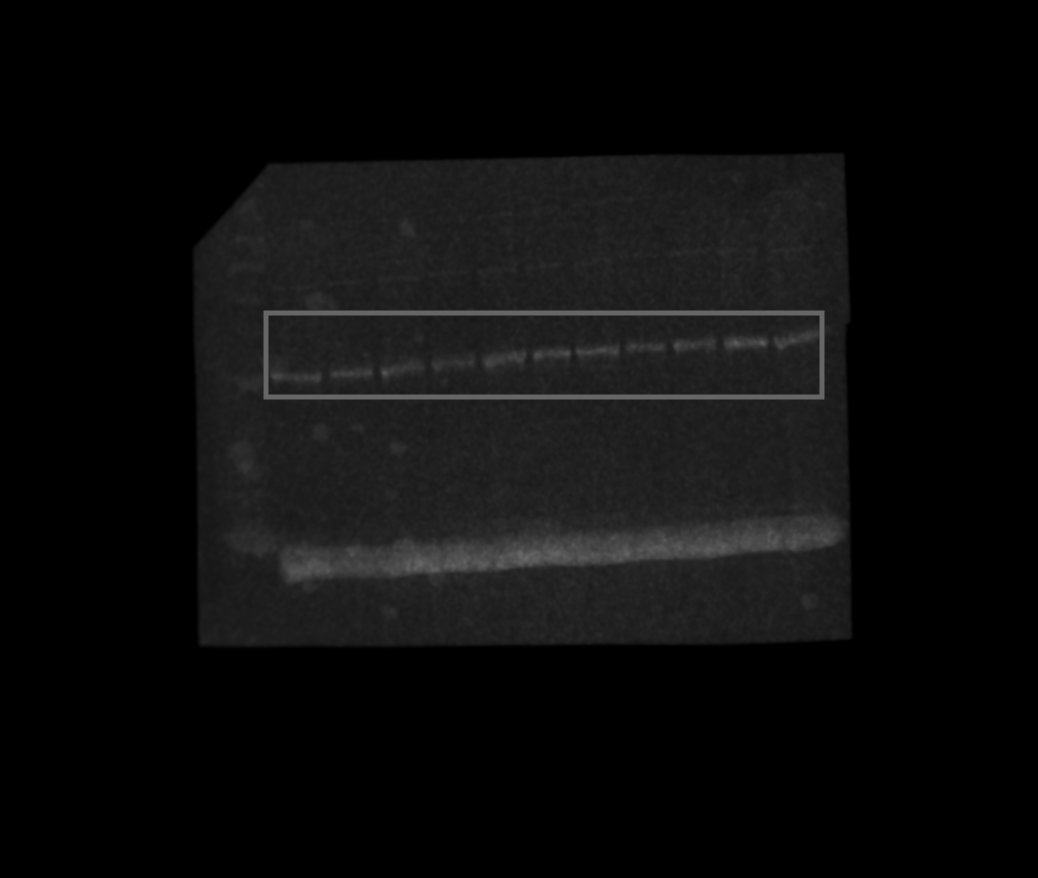

Supplement: Supplementary file 20 — Source Data [file 41467_2024_52215_MOESM20_ESM.zip › Source_data/FigS6A_western_3_GAPDH.TIF]

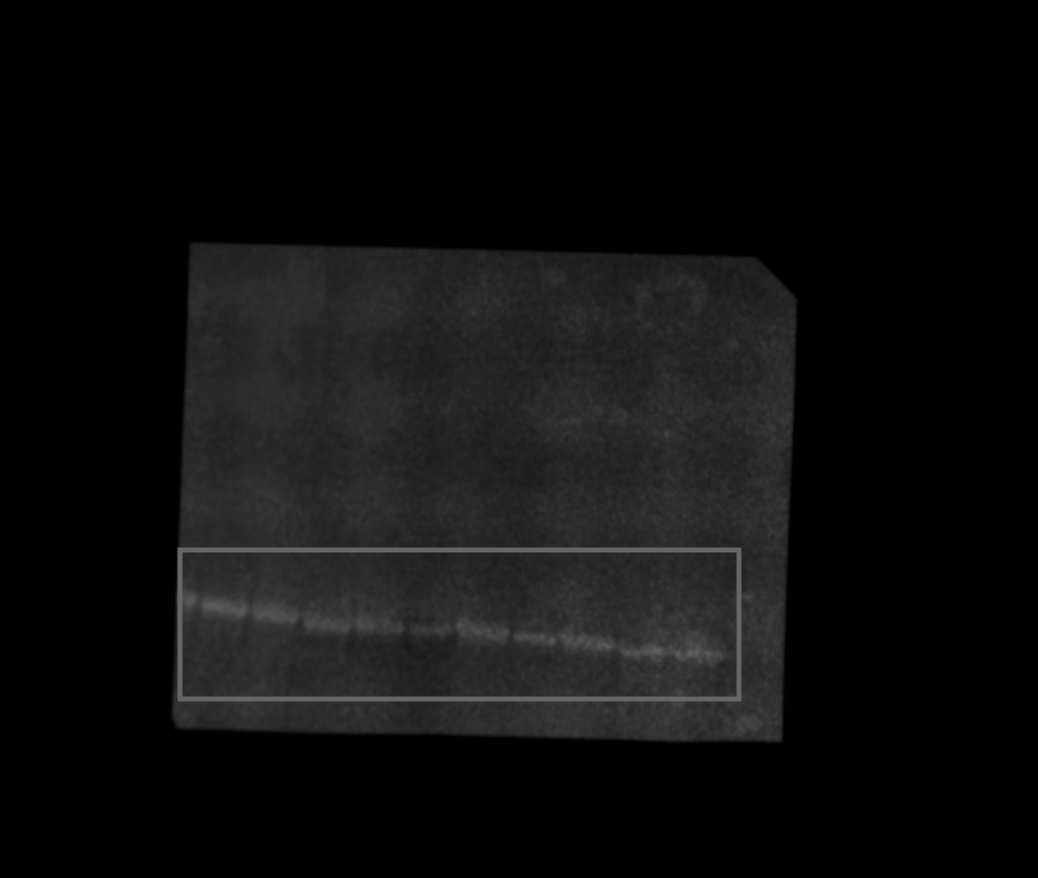

Supplement: Supplementary file 20 — Source Data [file 41467_2024_52215_MOESM20_ESM.zip › Source_data/FigS6A_western_6_Tubulin.TIF]

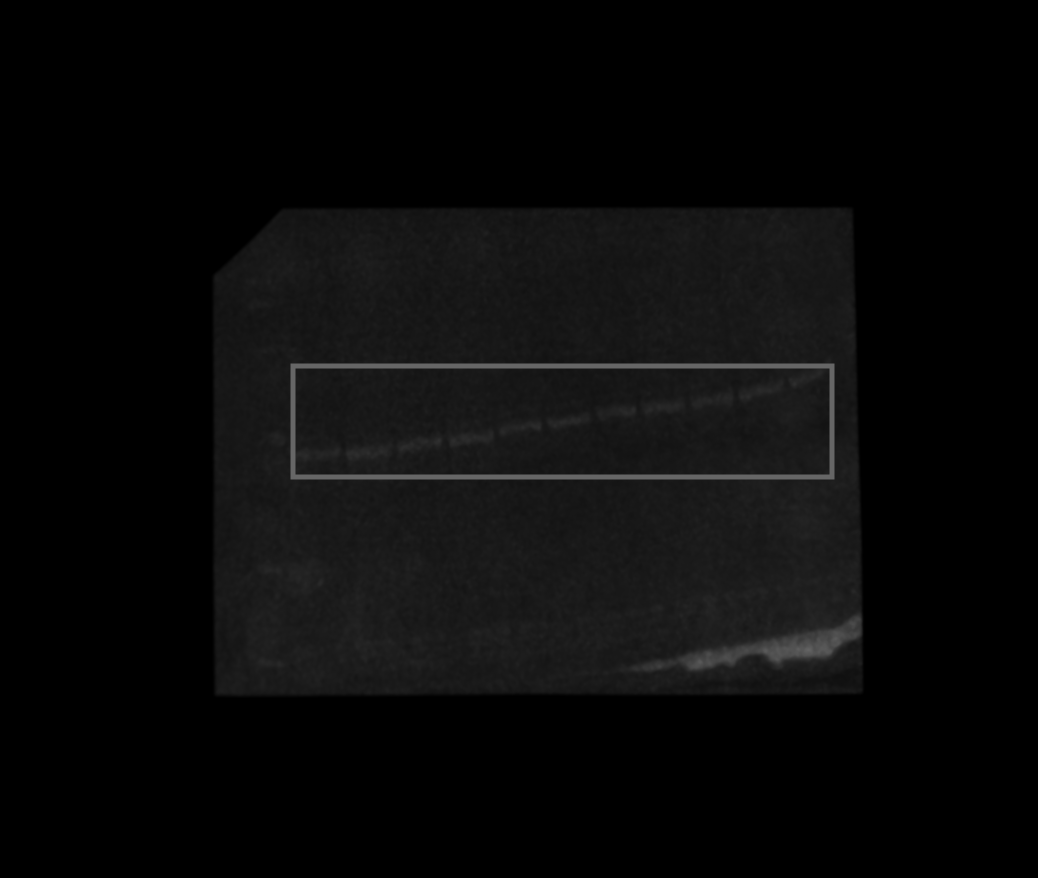

Supplement: Supplementary file 20 — Source Data [file 41467_2024_52215_MOESM20_ESM.zip › Source_data/FigS6A_western_5_GAPDH.TIF]

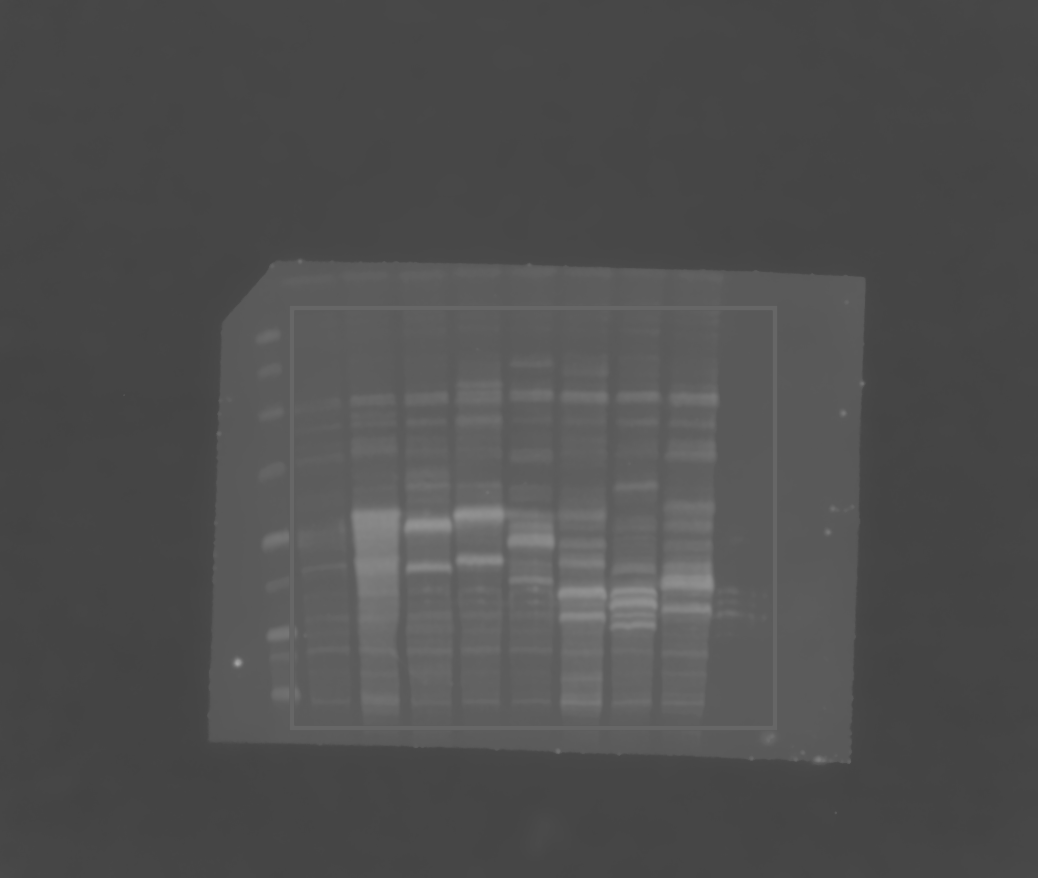

Supplement: Supplementary file 20 — Source Data [file 41467_2024_52215_MOESM20_ESM.zip › Source_data/FigS6A_western_4_HA.TIF]

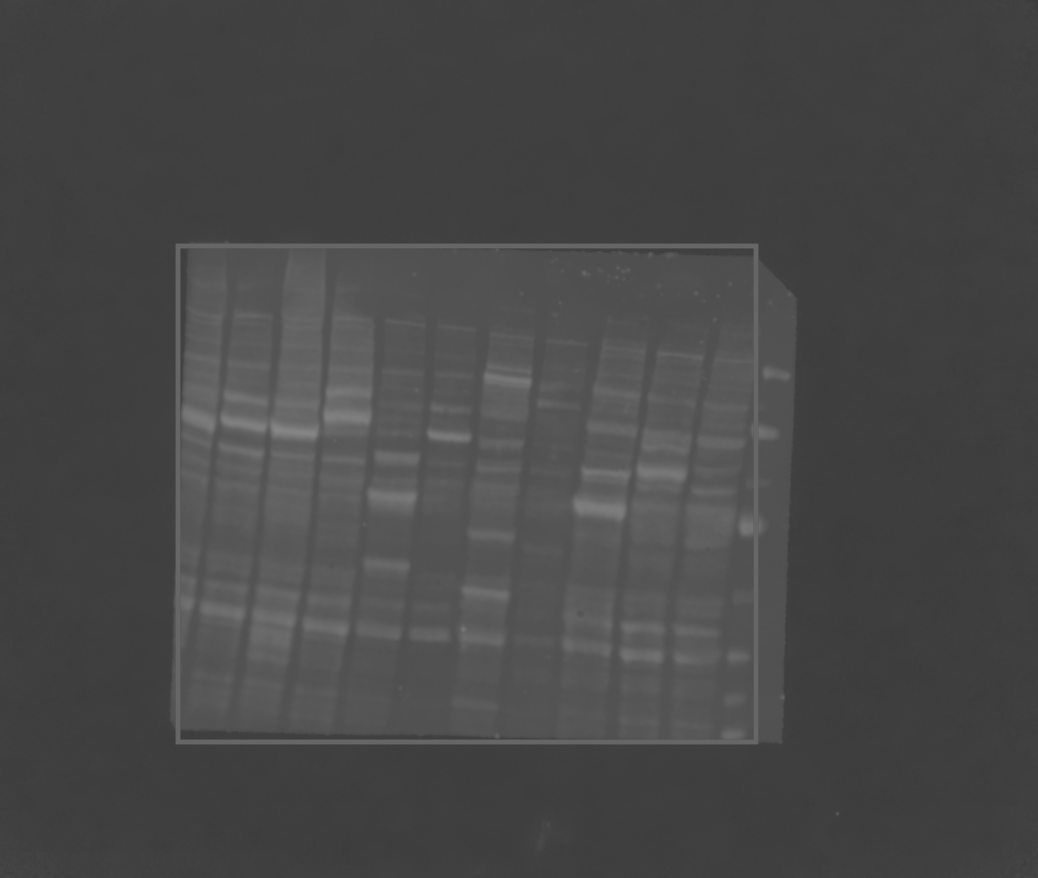

Supplement: Supplementary file 20 — Source Data [file 41467_2024_52215_MOESM20_ESM.zip › Source_data/FigS6A_western_6_HA.TIF]

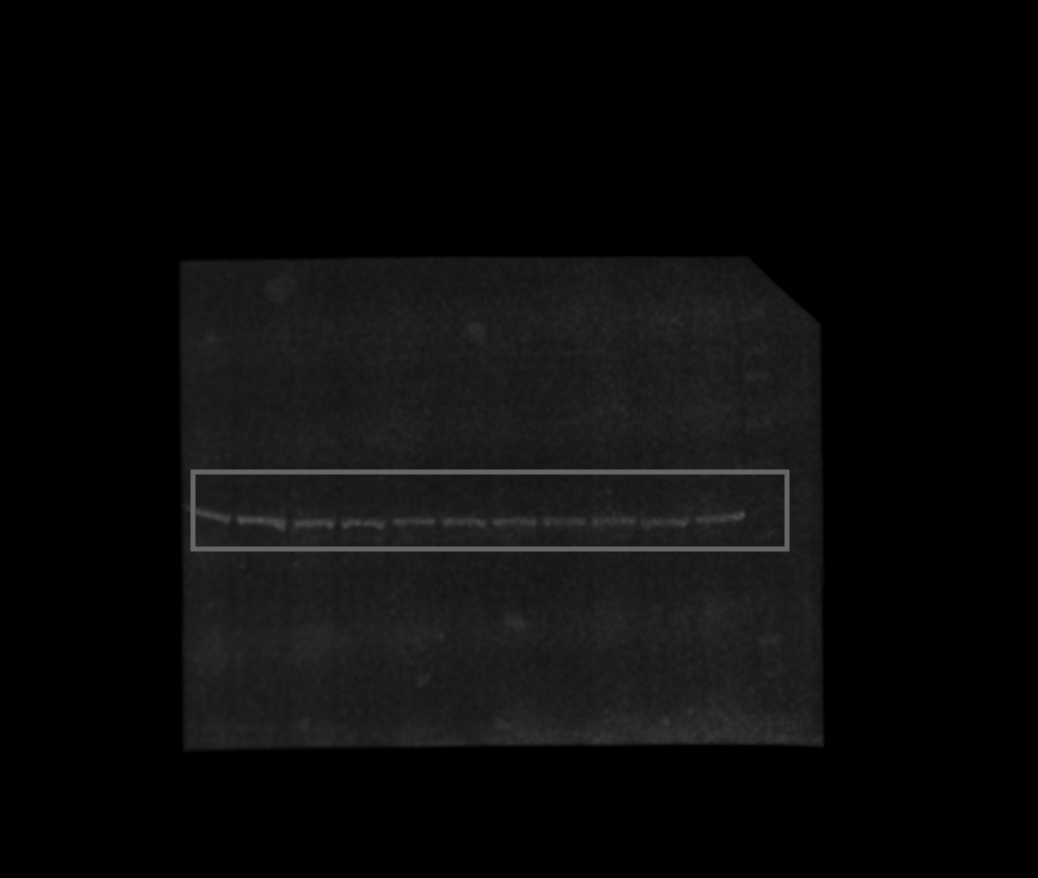

Supplement: Supplementary file 20 — Source Data [file 41467_2024_52215_MOESM20_ESM.zip › Source_data/FigS6A_western_2_Tubulin.TIF]

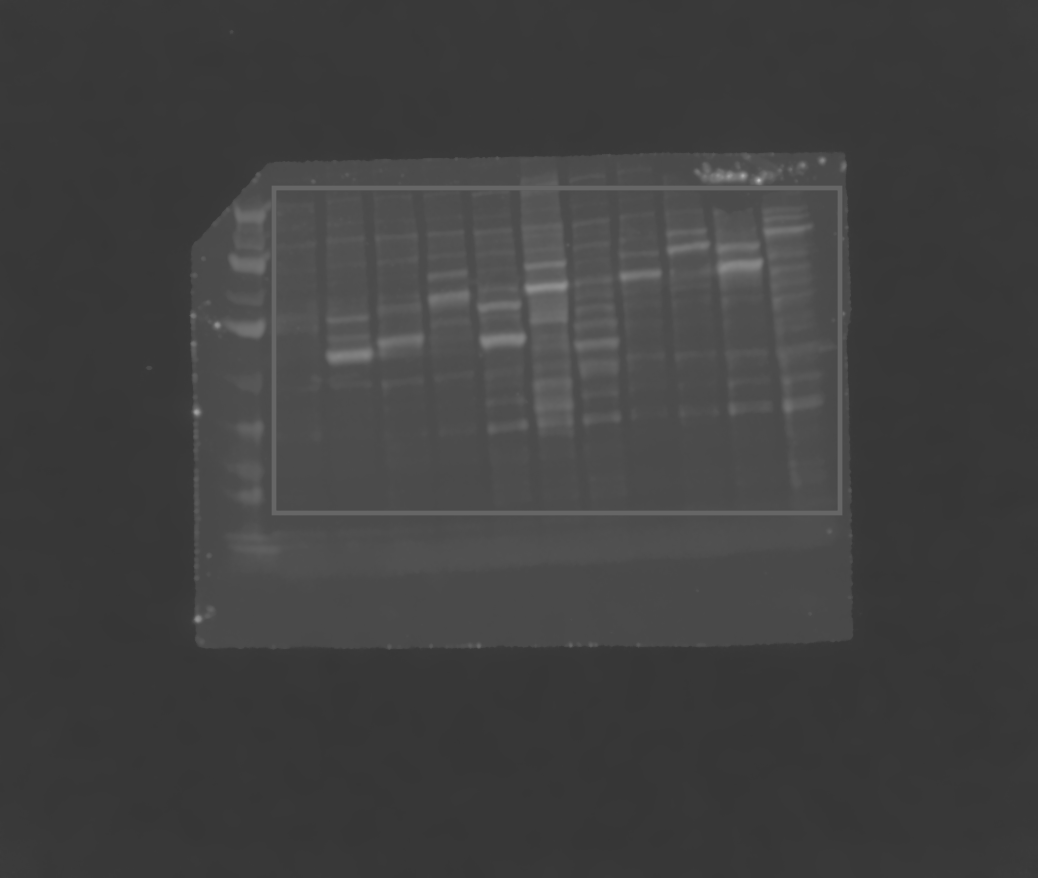

Supplement: Supplementary file 20 — Source Data [file 41467_2024_52215_MOESM20_ESM.zip › Source_data/FigS6A_western_3_HA.TIF]

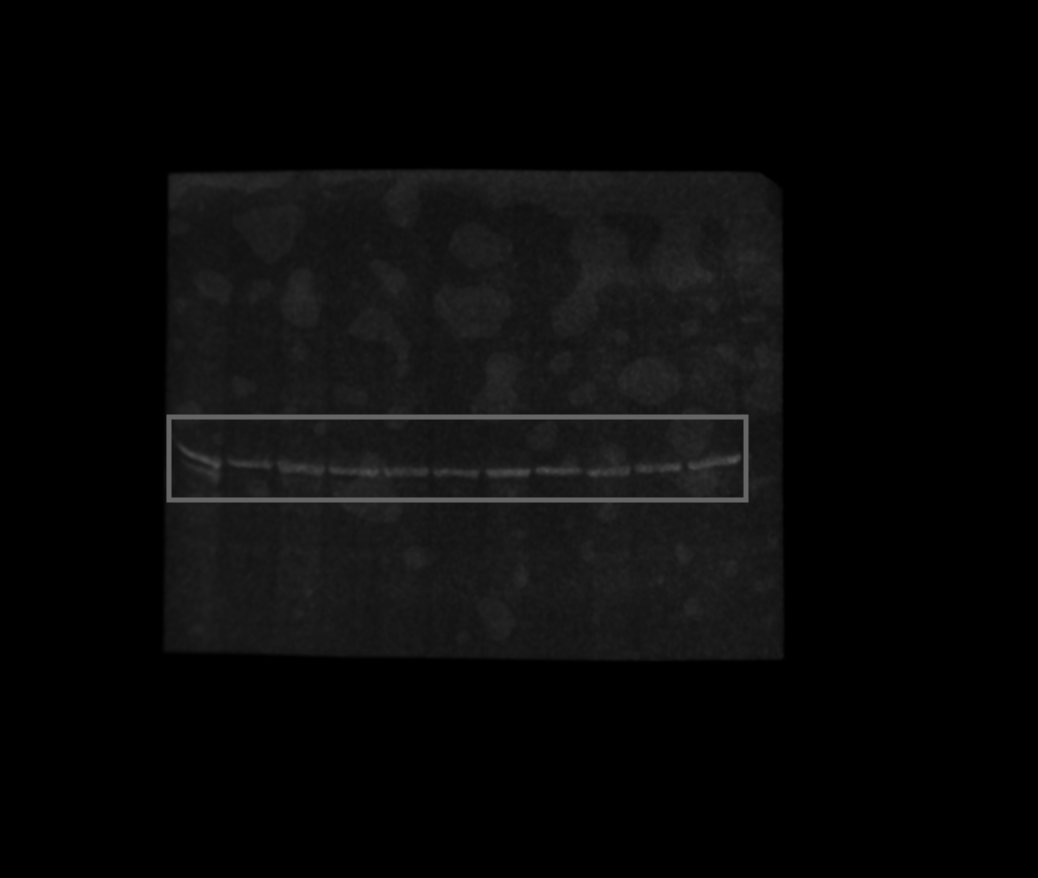

Supplement: Supplementary file 20 — Source Data [file 41467_2024_52215_MOESM20_ESM.zip › Source_data/FigS6A_western_7_Tubulin.TIF]

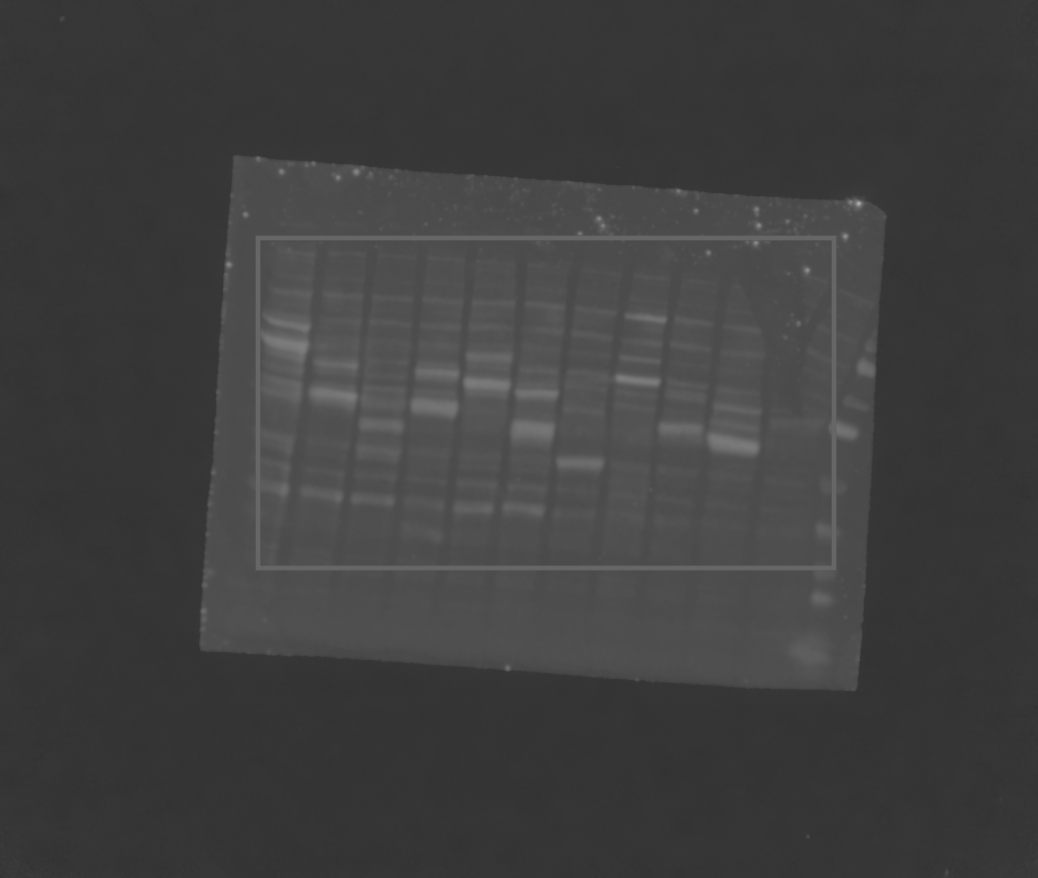

Supplement: Supplementary file 20 — Source Data [file 41467_2024_52215_MOESM20_ESM.zip › Source_data/FigS6A_western_1_HA.TIF]

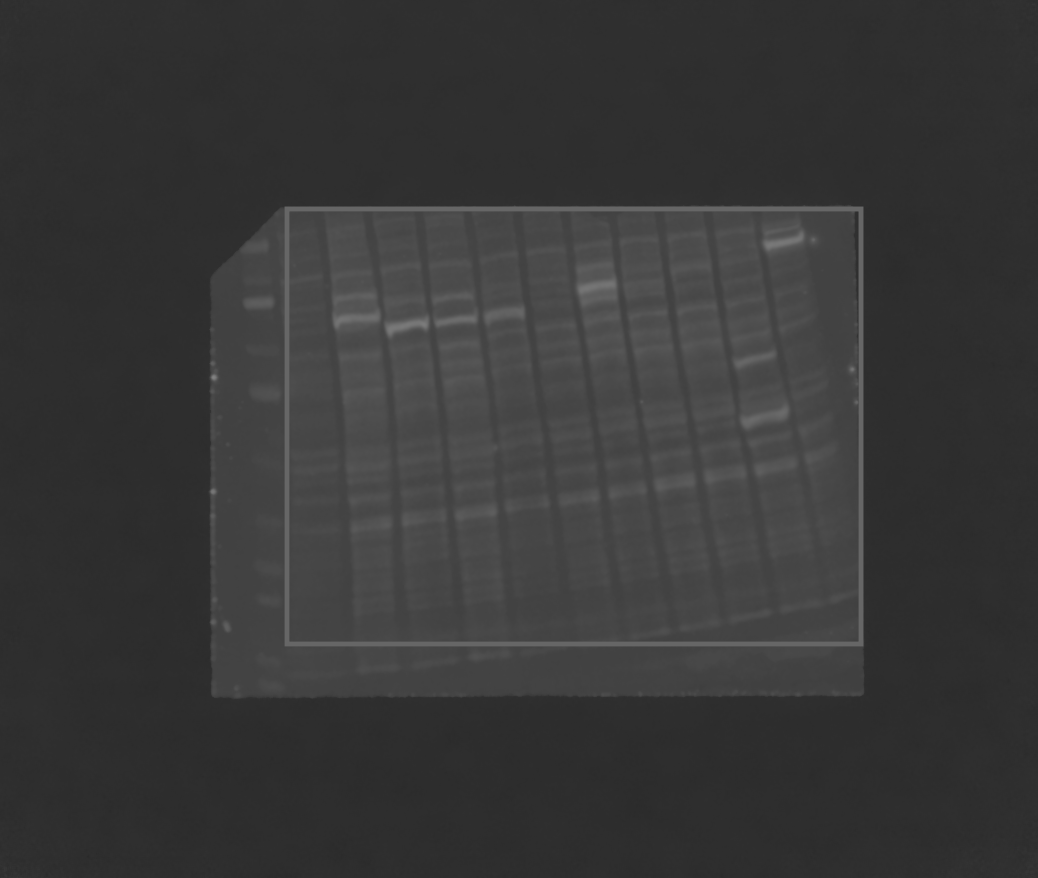

Supplement: Supplementary file 20 — Source Data [file 41467_2024_52215_MOESM20_ESM.zip › Source_data/FigS6A_western_5_HA.TIF]

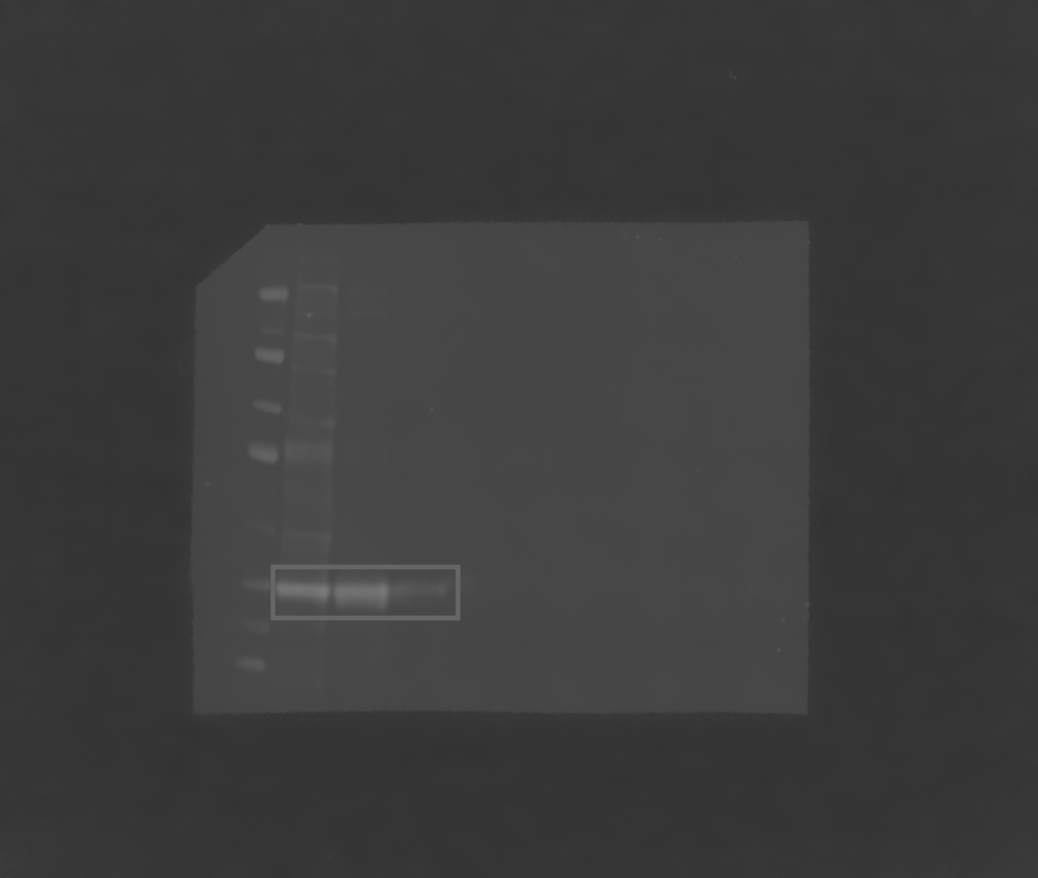

Supplement: Supplementary file 20 — Source Data [file 41467_2024_52215_MOESM20_ESM.zip › Source_data/FigS6B_western_eIF3I.TIF]

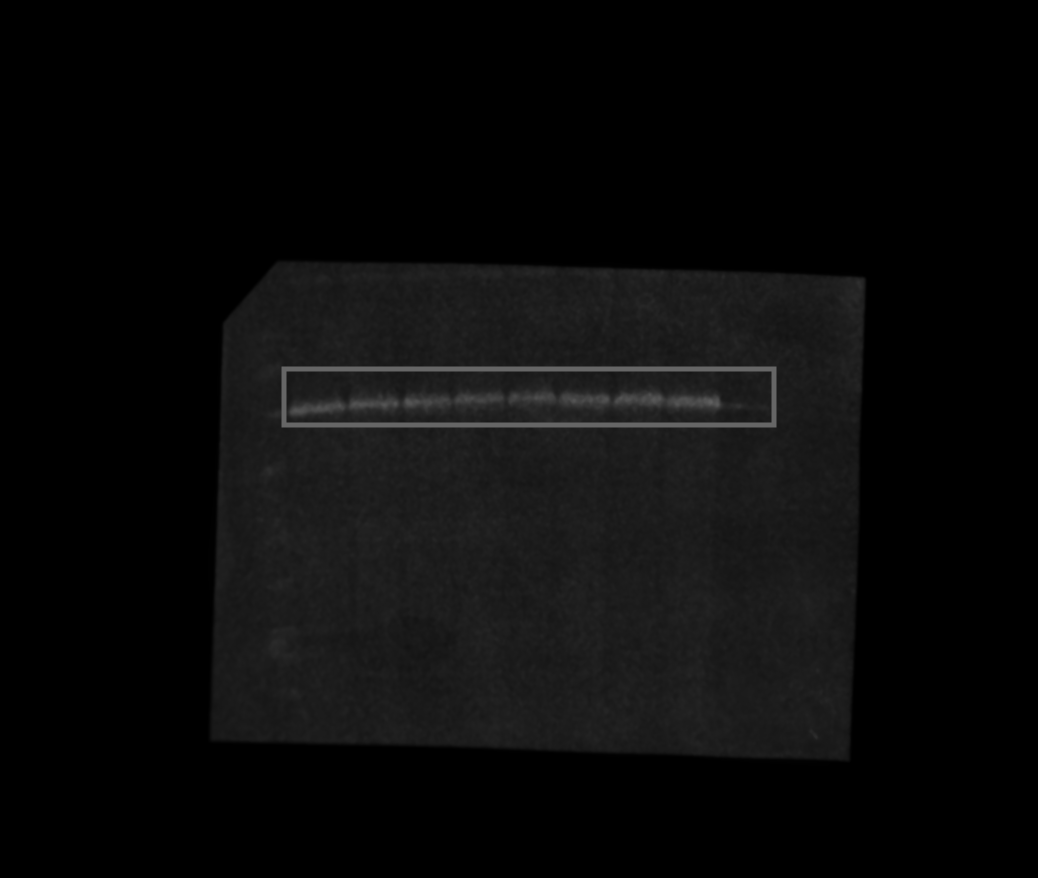

Supplement: Supplementary file 20 — Source Data [file 41467_2024_52215_MOESM20_ESM.zip › Source_data/FigS6A_western_4_Tubulin.TIF]

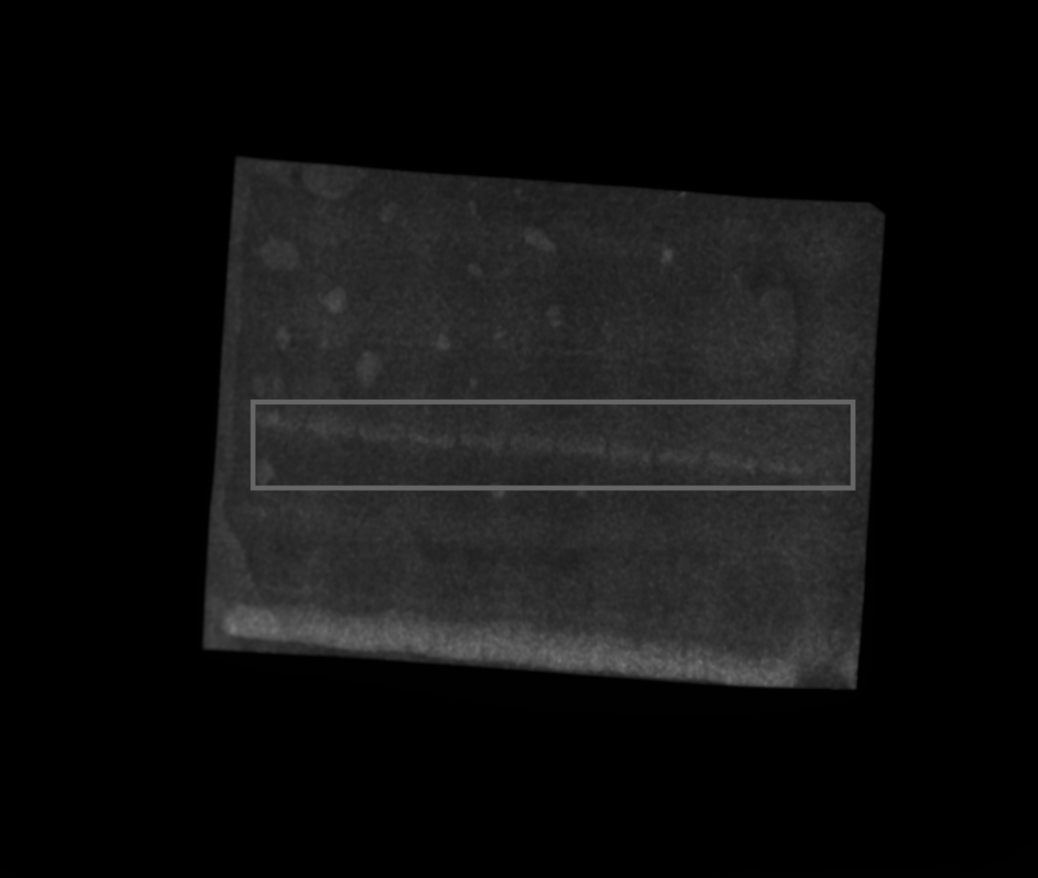

Supplement: Supplementary file 20 — Source Data [file 41467_2024_52215_MOESM20_ESM.zip › Source_data/FigS6A_western_1_Tubulin.TIF]

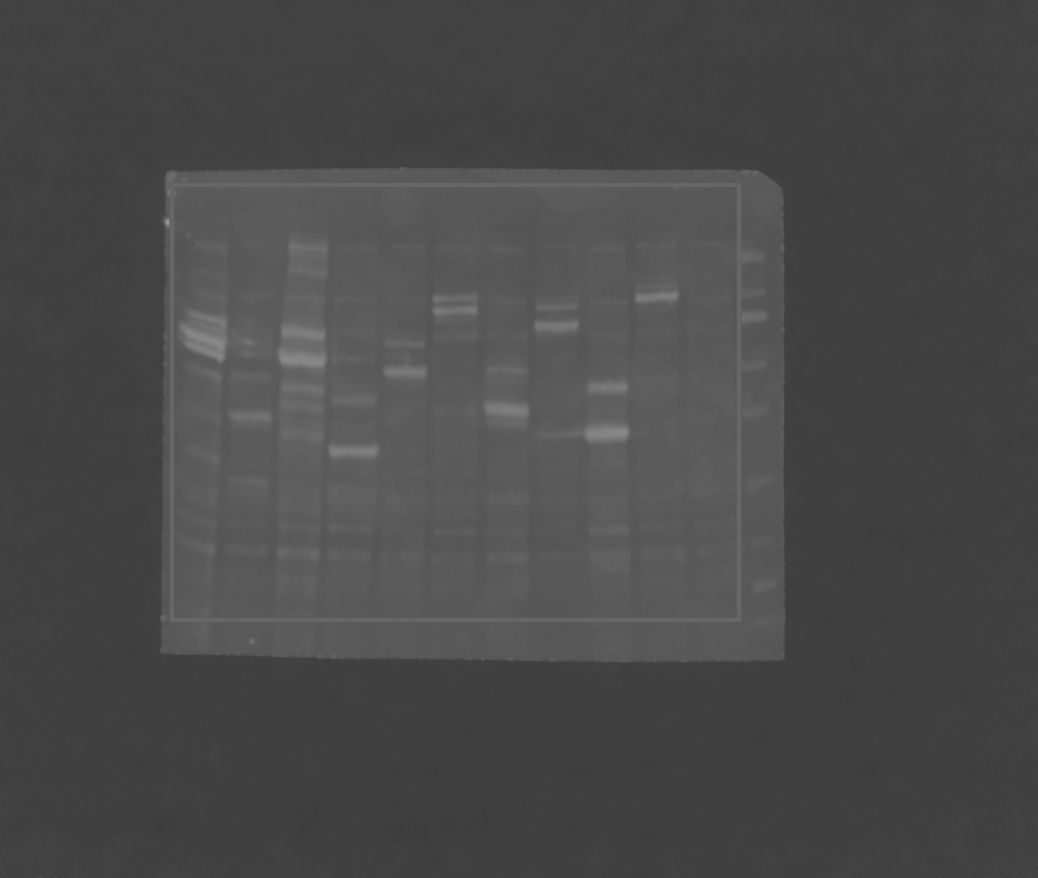

Supplement: Supplementary file 20 — Source Data [file 41467_2024_52215_MOESM20_ESM.zip › Source_data/FigS6A_western_7_HA.TIF]
